# De novo assembly of *Persea americana* cv. ‘Hass’ transcriptome during fruit development

**DOI:** 10.1186/s12864-019-5486-7

**Published:** 2019-02-06

**Authors:** Cristian Vergara-Pulgar, Karin Rothkegel, Mauricio González-Agüero, Romina Pedreschi, Reinaldo Campos-Vargas, Bruno G. Defilippi, Claudio Meneses

**Affiliations:** 1 Facultad de Ciencias de la Vida, Centro de Biotecnología Vegetal, Universidad Andres Bello, Avenida República 330, 8370035 Santiago, RM Chile; 2 Instituto de Investigaciones Agropecuarias, INIA-La Platina, Santa Rosa 11610, La Pintana, 831314 Santiago, RM Chile; 3 Escuela de Agronomía, Pontificia Universidad Católica de Valparaíso, Quillota, Chile; 4 FONDAP Center for Genome Regulation, Santiago, Chile

**Keywords:** RNA-Seq, Avocado, De novo transcriptome, Fruit development, Biomarkers

## Abstract

**Background:**

Avocado (*Persea americana* Mill.) is a basal angiosperm from the *Lauraceae* family. This species has a diploid genome with an approximated size of ~ 920 Mbp and produces a climacteric, fleshy and oily fruit. The flowering and fruit set are particularly prolonged processes, lasting between one to three months, generating important differences in physiological ages of the fruit within the same tree. So far there is no detailed genomic information regarding this species, being the cultivar ‘Hass’ especially important for avocado growers worldwide. With the aim to explore the fruit avocado transcriptome and to identify candidate biomarkers to monitore fruit development, we carried out an RNA-Seq approach during 4 stages of ‘Hass’ fruit development: 150 days after fruit set (DAFS), 240 DAFS, 300 DAFS (harvest) and 390 DAFS (late-harvest).

**Results:**

The ‘Hass’ de novo transcriptome contains 62,203 contigs (x̅=988 bp, N50 = 1050 bp). We found approximately an 85 and 99% of complete ultra-conserved genes in eukaryote and *plantae* database using BUSCO (Benchmarking Universal Single-Copy Orthologs) and CEGMA (Core Eukaryotic Gene Mapping Approach), respectively. Annotation was performed with BLASTx, resulting in a 58% of annotated contigs (90% of differentially expressed genes were annotated). Differentially expressed genes analysis (DEG; with False Discovery Rate ≤ 0.01) found 8672 genes considering all developmental stages. From this analysis, genes were clustered according to their expression pattern and 1209 genes show correlation with the four developmental stages.

**Conclusions:**

Candidate genes are proposed as possible biomarkers for monitoring the development of the ‘Hass’ avocado fruit associated with lipid metabolism, ethylene signaling pathway, auxin signaling pathway, and components of the cell wall.

**Electronic supplementary material:**

The online version of this article (10.1186/s12864-019-5486-7) contains supplementary material, which is available to authorized users.

## Background

Avocado (*Persea americana* Mill.) belongs to the *Lauraceae* family, one of the oldest and largest flowering plant families that includes over 50 genera [1]. Due to its nutritional properties, avocado is one of the major fruit crops worldwide and its commercial production is based on selections within three races and hybrids between them: the Mexican race *P. americana* var. *drymifolia*, the Guatemalan race *P. americana* var. *guatemalensis* and the West Indian race *P. americana* var. *americana* [2]. Depending on the race and variety, the development of the avocado fruit may vary, but generally is characterized by a biphasic behavior: the first phase is fruit maturation and occur while the fruit is attached to the tree, this stage is pre-climacteric (on-tree storage) and is associated to low ethylene levels and respiration rate. Later, a second phase or climacteric peak begins after the detachment of the fruit and it is characterized by an increase in ethylene levels and respiration rate, resulting in ripening and softening [2–5].

Fruit development and ripening of avocado are unique, with a constant cell division and cell growth during development. While for other angiosperms, the cell division ceases followed by cell growth [6]. During development, one of the main characteristics of avocado is the large accumulation of oil fraction that is used as harvest and quality indexes, which can be up to 9–15% of oil in fresh weight, levels that are dependent on maturity stage and growing conditions in ‘Hass’ avocado [7–10]. Additionally, the mesocarp also accumulates high nutrient levels, which includes 6% of carbohydrates, 2% of proteins and vitamins E, C, B2, B12, B1, K and D [6, 11].

Despite the economic and nutritional importance of avocado fruit, genomic data and resources are scarce. In last years, the development of new sequencing platforms and bioinformatic tools has been critical to study non-model organisms that lack a reference genome at a low cost [12–14]. One of these platforms, RNA sequencing (RNA-seq), is useful to find functional elements of the genome and to understand different developmental stages and tissues. Thus, the transcriptomic analysis allows us to identify candidate genes that could be associated with phenotype and biological processes [15, 16].

Transcriptomic changes during ripening stages (pre-climacteric; climacteric and post-climacteric) have been previously studied in the Mexican avocado var. *drymifolia* [17]. Regarding to ‘Biological process’GO terms, the authors reported significant changes in transcripts related to major biological changes like: ‘monosaccharide metabolic process’, ‘carbohydrate catabolic process’, ‘cell wall macromolecule catabolic process’, ‘cellular lipid metabolic process’ and ‘secondary metabolic process’, indicating that the most important changes associated to ripening include color (loss of green), firmness (cell wall degrading activities), taste (increase in sugars) and flavor (increase of volatile compounds) [17]. Furthermore, Kilaru and colleagues [18] studied the mesocarp tissue of avocado fruit at five stages to generate temporal transcriptomic data in order to associate expression patterns of lipid biosynthesis genes with oil accumulation and fatty acid content. However, molecular markers or biomarkers associated with fruit development and/or harvest date for marker-assisted selection are not yet available.

To further elucidate some of the molecular factors involved in the regulation of fruit development of the economically important ‘Hass’ avocado variety, we conducted a de novo assembly of the transcriptome from the developing mesocarp at 150, 240, 300 (first harvest) and 390 (second harvest) days after fruit set (DAFS) using HiSeq 2500 (Illumina) platform. From this data, our aim was to explore the fruit avocado transcriptome during development in order to identify candidate genes that could be further characterized as potential biomarkers to monitor fruit development. Finally, to confirm RNA-seq data and de novo assembly, genes related to lipid metabolism were analyzed through quantitative Real Time PCR.

## Results

### Illumina sequencing and assembly of the avocado de novo transcriptome

In order to obtain insights of the dynamics of the ‘Hass’ avocado transcriptome during fruit development, RNA libraries were constructed and sequenced at four developmental stages (150, 240, 300 and 390 DAFS) using a HiSeq 2500 (Illumina) sequencer. Sequencing yielded a total of 261,409,226 pair-end reads (2 × 150 bp long) considering three biological replicates for each developmental stage (12 libraries). Raw data were submitted and they are available at the NCBI database (BioProject: PRJNA483022). The reads were trimmed to remove low quality reads, obtaining a total of 260,628,748 processed reads that were used as input to assemble the transcriptome with Trinity software (see Additional file 1). From the processed reads, a total of 62,203 contigs were assembled with an average size of 987 bp an N50 of 1050 bp and 62,167 predicted genes (Table 1). From CEGMA, a 99% of complete genes were found, meantime for BUSCO a total of 972 complete and single copy genes (84.1%) were found (Table 1).

**Table 1:** De novo transcriptome metrics. Metrics of quality obtained from de novo assembly during fruit development in *P. Americana* cv. ‘Hass’ are shown. The entire read raw data from four fruit developmental stages and three biological replicates were used for the assembly

Metric	
Total Contigs	62,203
Trinity “Genes”	62,167
N10 (bp)	2594
N30 (bp)	1511
N50 (bp)	1050
Largest contig length (bp)	11,212
Smallest contig length (bp)	401
Average contig length (bp)	987
GC (%)	41.57
Total nucleotides	61,399,929
CEGMA Score (%)	C^1^: 99; F^2^: 1; M^3^: 0
BUSCO Score (%)	C^1^: 85; F^2^: 8; M^3^: 7
TransRate Score	0.12/0.14

()^+^: BUSCO and CEGMA dataset used contains: C^1^: Complete genes found from the dataset, F^2^: Fragmented genes found from the dataset and M^3^: Missing genes. CEGMA dataset used contains 247 eukaryotic proteins and BUSCO dataset used contains 957 plant specific proteins

**Fig. 1:**
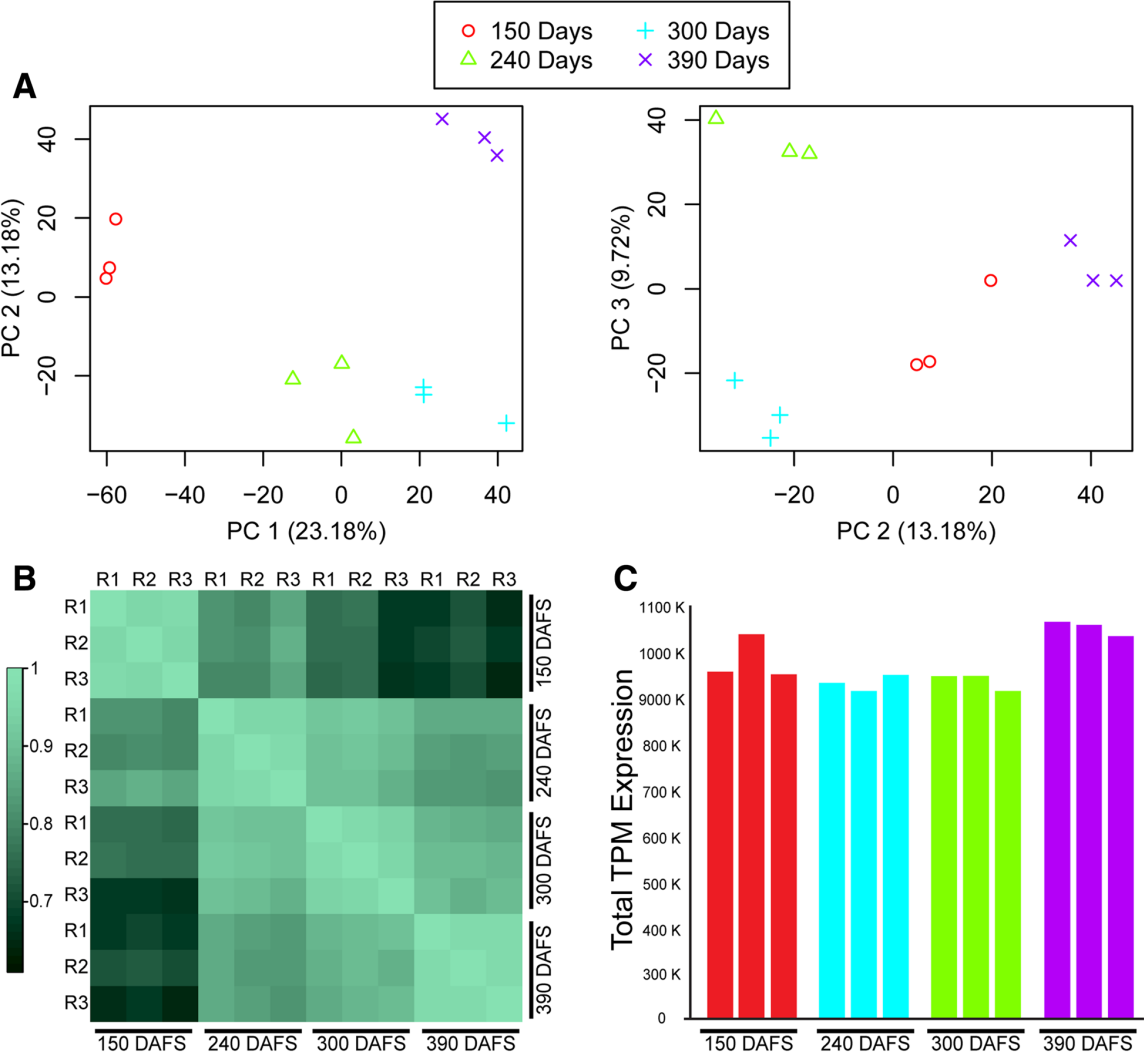
Overall gene expression during fruit developmental in *P. americana* by RNA-seq strategy. **a** PCA of the genes global expression from four development stages (150, 240, 300 and 390 days after fruit set or DAFS) and their three biological replicates (R1, R2 and R3). **b** Similarity heatmap based on the Pearson correlation among fruit developmental stage and biological replicates (**c**) Total TPM-TMM normalized expression for each stages and biological replicates

In order to estimate the variability of gene global expression among fruit development stages and their biological replicates, the PCA explained up to 36.4% of the variability in PC1 and PC2 (Fig. 1a), while in PC2 and PC3 explained 22.90%. The PCA graph results displayed consistency among biological replicate samples and stages of fruit development. The first component explained 23.18% of variance and separates 150 DAFS from 390 DAFS. On the other hand, PC3 (9.72%) achieve the separation of 240 DAFS from 300 DAFS. In addition, Pearson correlation matrix shows high similarity among biological replicates, which is consistent with PCA (Fig. 1b). In both cases (PCA analysis and correlation matrix), homogeneity is shown between the biological replicates and, on the other hand, there are significant changes in the transcriptome during fruit development. To compare transcriptomic data among samples, normalization of the reads through transcripts per million (TPM) was performed. In relation with the overrall gene expression, we obtained an homogeneous level of gene expression (1,000,000 – 1,100,000 TPM) among the four developmental stages and biological replicates (Fig. 1c).

### Functional annotation and global GO terms of the de novo transcriptome

**Fig. 2:**
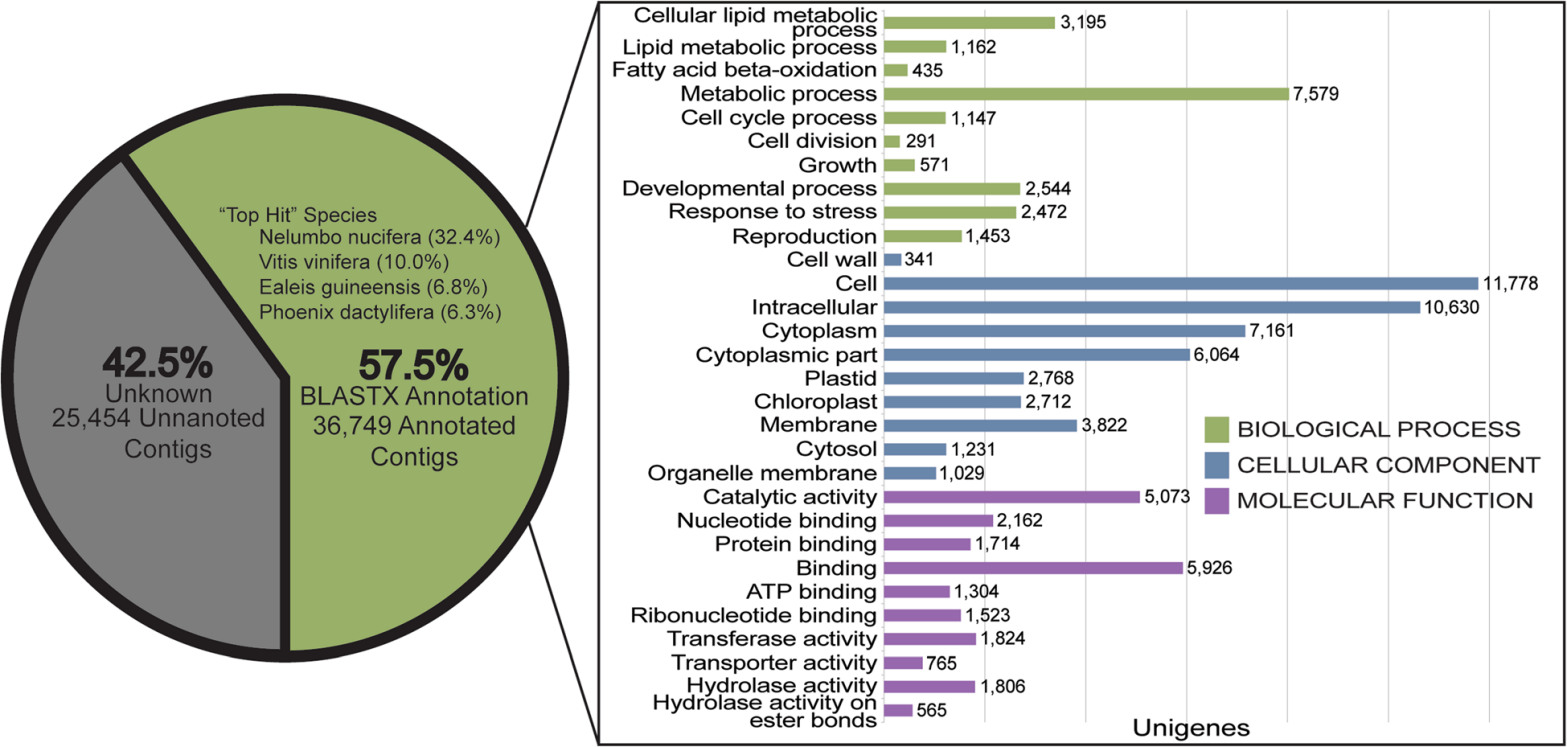
Annotation and relevant GO terms of the de novo transcriptome assembled. 36,749 of 62,203 contigs were annotated with BLASTx. The top hit species were *Nelumbo nucifera* (32.4%), *Vitis vinifera* (10.0%), *Elaeis guineensis* (6.8%) and *Phoenix dactylifera* (6.3%). Top 10 relevant level 1 GO categories from unigenes are displayed for biological process (BP) on green, cellular component (CC) on blue and molecular function (MF) on purple

To annotate the 62,203 contigs, we searched for reference sequences using BLASTx (https://blast.ncbi.nlm.nih.gov/Blast.cgi), against the NCBI database. Among the total assembled contigs 36,749 transcripts (57.5%) had a significant hit (Fig. 2). From these, a 32.4% were annotated using BLASTx obtaining as best hits *Nelumbo nucifera*, followed by *Vitis vinifera* (10.0%), *Elaeis guineensis* (6.8%) and *Phoenix dactylifera* (6.3%) (see Additional file 2). The 36,749 expressed genes were also used for Gene Ontology (GO) analysis and associated to different GO terms (Fig. 2). The GO terms were classified, and transcripts were assigned to biological process (GO:0008150), molecular function (GO:0003674) and cellular component (GO:0005575) categories. In the biological process category, the two largest groups were ‘Metabolic process’ and ‘Cellular lipid metabolic process’; meanwhile ‘Cell’ and ‘Intracellular’ are the largest groups for the cellular component category. For the molecular function category, ‘Binding’ and ‘Catalytic activity’ accounted for most of the annotated genes (Fig. 2).

### GO classification of DEGs and global differential expression analysis during development

In order to globally understand the development process, differentially expressed genes were used to perform GO analyzes, using the comparisons of 150 DAFS vs 240 DAFS, 240 DAFS vs 300 DAFS and 300 DAFS vs 390 DAFS. For the first comparison (150 DAFS vs 240 DAFS), we obtained a significant enrichment (from 35.71 to 45.83%) of transcripts involved in the processes of ‘lipid localization’ (GO:0010876), ‘tubulin complex’ (GO:0045298), ‘fatty-acid synthase activity’ (GO:0004312) and ‘acyl carrier activity’ (GO:0000036) (Additional file 3). When comparing 240 DAFS and 300 DAFS (first harvest), we can observe the overexpression (from 7.06 to 9.43%) of genes related to ‘response to hydrogen peroxide’ (GO:0042542) and ‘response to reactive oxygen species’ (GO:0000302) (Additional file 3). Finally, for the third comparison (300 DAFS vs 390 DAFS), genes related to ‘post-embryonic morphogenesis’ (GO:0009886) were overexpressed with a 14.29% of frequency (Additional file 3). Additionally, we identified transcription factors among the DEG for all the conditions during fruit development (Additional file 4).

**Fig. 3:**
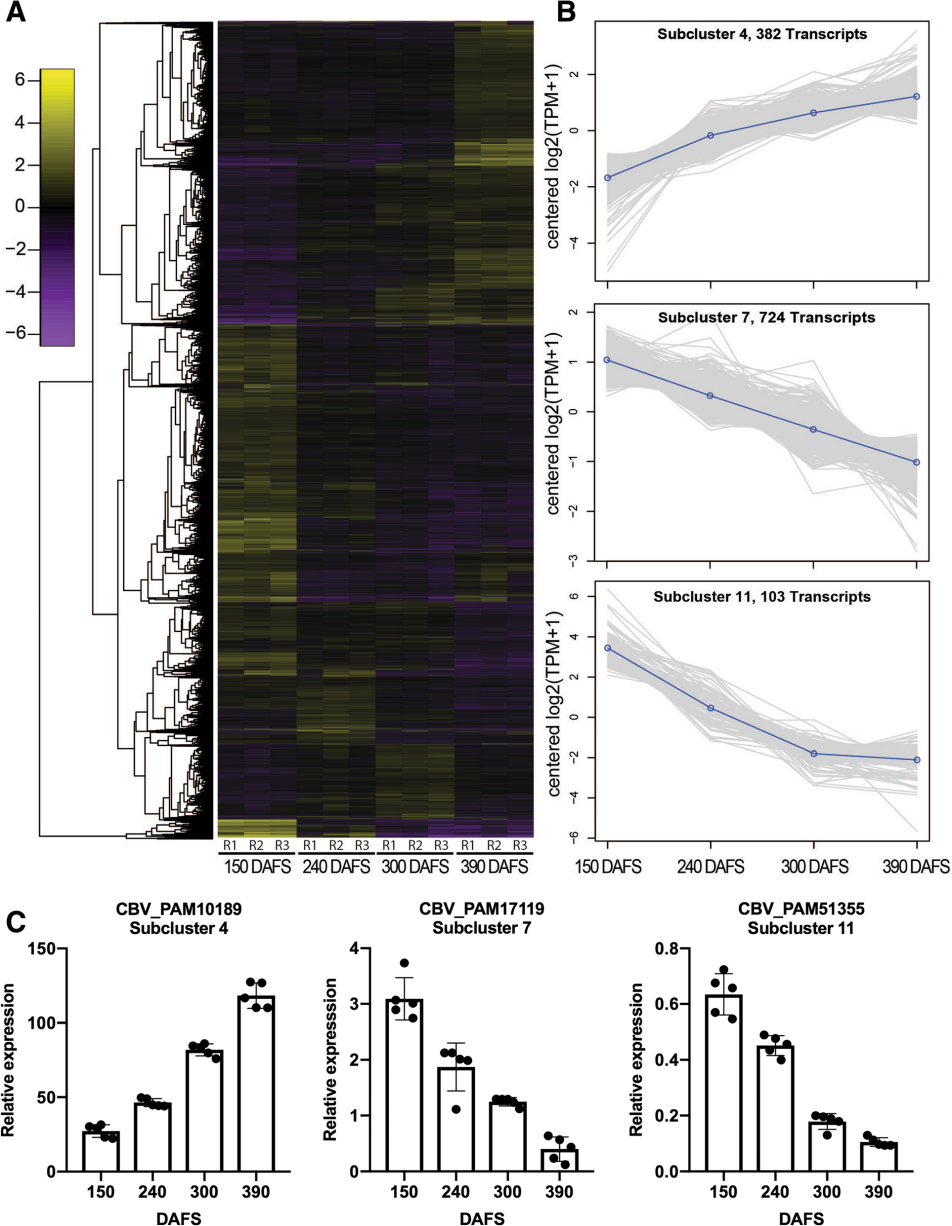
Differentially Expressed Genes analsyis. Profiling expression of DEG in *P. americana* cv. ‘Hass’(**a**) Heatmap of DEGs (FC ≥ 1 and FDR ≤ 0.01) across all four developmental stages, resulting in 8672 differentially expressed genes (DEGs). Y-axis corresponds to the different genes used as input information, X-axis shows the different conditions with their respective biological replicates. The color key represents the median centered log2 TMM-normalized TPM values. **b** Subcluster Plots, three out of fifteen (K = 15) subclusters were used to find candidate genes because of their expression profiles. The X-axis shows the four developmental stages and the Y-axis is the median centered log2 expression. **c** Validation of gene expression by qPCR for selected genes from subclusters 4, 7 and 11: CBV_PAM10189 (subcluster 4), CBV_PAM17119 (subcluster 7) and CBV_PAM1355 (subcluster 11). The Y-axis displays relative expression levels to *PamActin* gene, while the X-axis indicates the development stage

On the other hand, in order to identify candidate genes involved in the development of avocado fruit, we compared samples from 150, 240, 300 and 390 DAFS considering three biological replicates using differential expression analysis. We obtained 8672 differentially expressed genes [FDR ≤0.01 and Fold Change (FC) ≥1] that revealed significant differences and were clustered according to their regulation pattern (Fig. 3a). Fifteen subclusters were obtained (K = 15), where subclusters 4, 7 and 11 contain a total of 1209 genes that show a correlation with the four developmental stages (Fig. 3b; see Additional file 5 and Additional file 6). Among the 1209 genes, 382 transcripts belonging to subcluster 4 increase their abundance between 150 DAFS and 390 DAFS. On the contrary, transcripts of subcluster 7 (724 genes) and 11 (103 genes), strongly decrease their abundance starting from 150 DAFS (Fig. 3b). Functional annotation of these transcripts was performed using the KEGG automatic annotation server, where pathways of ‘carotenoid biosynthesis’, ‘fatty acid biosynthesis’, ‘starch and sucrose metabolism’ and ‘phenylpropanoid biosynthesis’ were identified (Additional file 7). Furthermore, genes from each subcluster were selected as candidates for further analysis as possible biomarkers for fruit development and harvest date in avocado. In subcluster 4, selected genes that constantly increase their expression were related to lipid storage, carbohydrate metabolism, phenylpropanoid biosynthesis, intracellular signal transduction, metabolic process, ethylene signaling pathway, auxin signaling pathway and cytokinin metabolic process (Table 2). In subcluster 7, genes that constantly decrease their expression were associated to sugar transport, cell signaling, lipid catabolism, lipid metabolic process, pigment biosynthetic process and cell signaling (Table 2). For subcluster 11, genes that constantly decrease their expression were related to the fatty acid biosynthesis pathway and components of cell wall (Table 2). On the other hand, we validated one gene selected from each subcluster (4, 7 and 11) by qPCR as biological validation. Concomitant with the subclusters profile, we obtained the same pattern in the qPCR analysis (Fig. 3c).

**Table 2:** Differentially expressed genes (DEG) that constantly increase (subcluster 4) and decrease (subclusters 7 and 11) their transcript levels during fruit development stages in *P. americana* cv. ‘Hass’. Subclusters were selected from gene expression cluster analysis

^+^Contig ID	Gene ID	Contig annotation	Sub-cluster
CBV_PAM10189	AGT63296	Oleosin	4
CBV_PAM50790	XP_010938396	Beta-glucosidase 22-like	4
CBV_PAM74617	AFG26325	Cinnamoyl-CoA reductase	4
CBV_PAM23929	XP_007042920	UDP-glucosyltransferase 85A4	4
CBV_PAM2452	CAC81811	Putative chitinase 1	4
CBV_PAM11370	KHN19579	Auxin-induced protein 5NG4	4
CBV_PAM38155	XP_010279539	Zeatin O-glucosyltransferase-like	4
CBV_PAM3819	XP_010261262	Ethylene-responsive transcription factor RAP2–4-like	4
CBV_PAM37547	XP_010929602	Bidirectional sugar transporter SWEET14-like	7
CBV_PAM51147	XP_010023633	Protein RALF-like 32	7
CBV_PAM51593	XP_010251703	Neutral ceramidase-like	7
CBV_PAM17119	ALG05139	Polyphenol oxidase	7
CBV_PAM55044	XP_010910148	Polyphenol oxidase	7
CBV_PAM52108	XP_010924766	Neutral ceramidase-like isoform X2	7
CBV_PAM11012	XP_010270351	ABC transporter G family member 5-like	7
CBV_PAM38418	XP_010261893	LRR receptor-like serine/threonine-protein kinase	7
CBV_PAM25422	XP_010263506	Probable serine/threonine-protein kinase	7
CBV_PAM1216	XP_012475944	14 kDa proline-rich protein DC2.15-like	11
CBV_PAM51355	AAL23676	Delta-12 fatty acid desaturase	11
CBV_PAM51992	XP_012488766	14 kDa proline-rich protein DC2.15-like	11
CBV_PAM1011	XP_010675675	Fatty acid desaturase 4, chloroplastic	11
CBV_PAM10195	XP_008811043	Proline-rich protein 4	11
CBV_PAM64888	XP_009395434	Probable polygalacturonase	11
CBV_PAM10256	XP_010047989	36.4 kDa proline-rich protein-like	11

()^+^: Codes used for the de novo transcriptome trinity genes

### Validation of DEGs by quantitative real-time PCR (qRT-PCR)

**Fig. 4:**
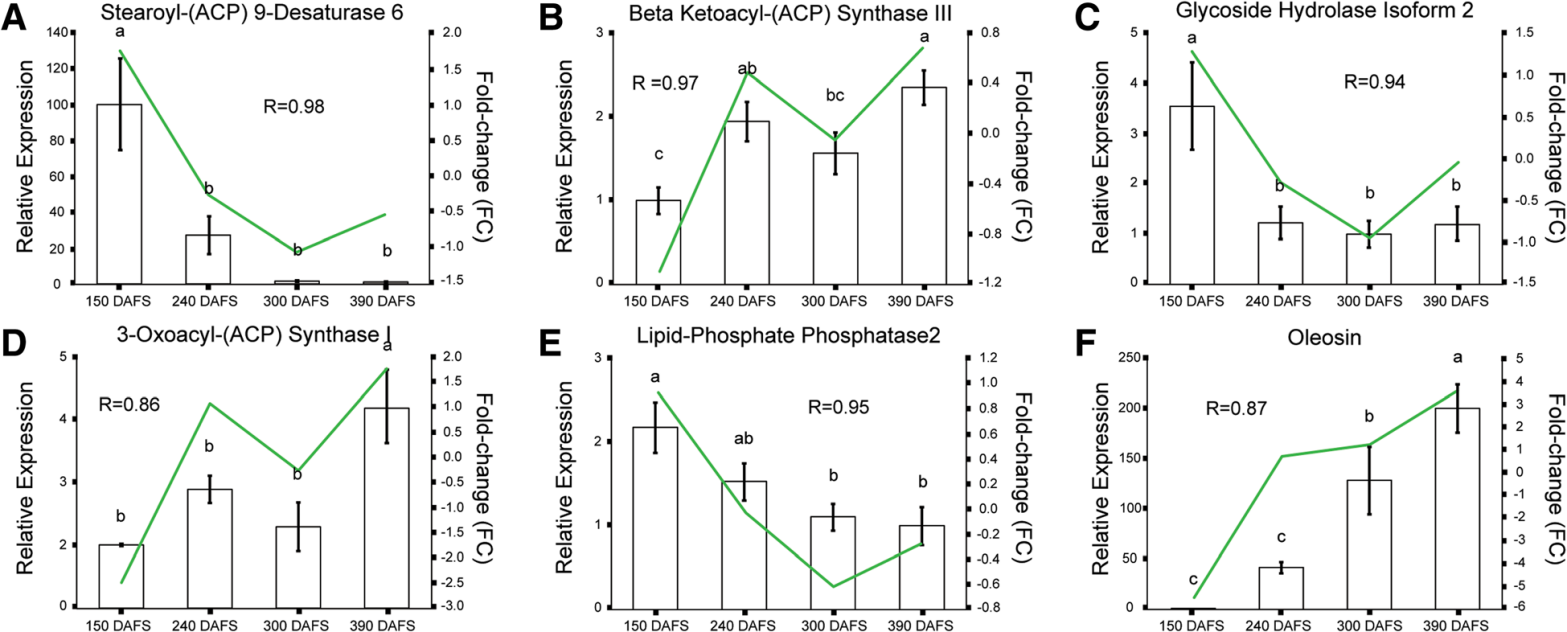
Correlation between qRT-PCR and in silico gene expression. The figure shows six transcripts analyzed this study: (**a**) Stearoyl-(ACP) 9-desaturase 6, (**b**) Beta-ketoacyl-ACP Synthase III, (**c**) Glycoside Hydrolase Isoform 2, (**d**) 3-Oxoacyl-(ACP) Synthase I, (**e**) Lipid Phosphate Phosphatase 2 and (**f**) Oleosin. The left Y axis displays the normalized relative expression levels to *PamActin* gene, while the right Y axis shows the FC values from the differential expression analysis, X axis indicates the development stage. R indicates the spearman correlation coefficient between in vitro and in silico expression values

To validate the de novo assembly and transcriptomic data, genes related to lipid metabolism, fatty acid biosynthesis and storage were used to analyze through qRT-PCR (Fig. 4). Therefore, the expression analysis for Stearoyl-(ACP) 9-Desaturase 6, Beta Ketoacyl-(ACP) Synthase III, Glycoside Hydrolase Isoform 2, 3-Oxoacyl-(ACP) Synthase I, Lipid-Phosphate Phospatase 2 and Oleosin were consistent with the transcriptomic data (Fig. 4). The Pearson correlation (*r* > 0.85) showed that there is a positive correlation between the two methods, validating the transcriptome analysis (Fig. 4; see Additional file 8).

## Discussion

### The de novo assembly and functional annotation of the avocado transcriptome

In this study we report the de novo assembly of the ‘Hass’ avocado transcriptome to identify candidate genes that correlate their RNA levels with different developmental stages (150, 240, 300 and 390 DAFS), allowing their future study as potential biomarkers for monitoring developmental stage and harvest date. In the last years, the development of new sequencing technologies and informatic tools, together with the available data of avocado form previous studies, has helped to improve assemblies and therefore, to better understand biological and molecular processes. In this work, from a short-read assembly we could obtain 62,203 contigs and 62,167 genes using Trinity (Table 1), while a previous study of the ‘Hass’ transcriptome reported a total of 134,329 contigs with a short-read assembly and another study of Mexican avocado obtained 83,650 contigs using a hybrid assembly (long reads + short reads) [17, 18]. Thus, in the present study we obtained an improved short-read assembly (62,203 contigs) with 85–99% of complete genes, providing with additional data that can contribute to the characterization of avocado development.

Transcriptomic analysis has been fundamental for the characterization and discovery of new molecular insights for biological processes like fruit development and ripening. In the plant model tomato, a spatiotemporal resolved transcriptome of tomato fruit revealed that the ripening program comprises gradients of gene expression from internal tissues to outward as maturation progressed [16, 19]. On the other hand, for non-model crops, like hami melon (*Cucumis melo*), sweet orange (*Citrus sinensis*), eggplant (*Solanum melongena*) and sweet cherry (*Prunus avium*), this platform has been critical to better understand the development process without a reference genome [20–23]. For *P. americana*, the normalized transcriptomic data was classified according to the transcript profile across the different developmental stages (Fig. 1). In this case, the PC could group the three biological replicates together and separate 150 DAFS from 390 DAFS and 240 DAFS from 300 DAFS (Fig. 1a). Together with this, the correlation analysis shows a decreasing similarity between 150 DAFS and 240, and 300 and 390 DAFS (Fig. 1b), reflecting the results obtained by PCA and indicating changes in gene expression during fruit development.

To annotate and to identify similarities between avocado transcripts and known plant proteins, we used BLASTx against the non-redundant NCBI database and obtained that 57.5% of total transcripts (36,749 out of 62,203 contigs) have similarity with annotated proteins (Fig. 2; Additional file 2). These genes were associated to GO terms, where the most represented groups from the biological process category corresponded to ‘metabolic’ and ‘cellular lipid’ processes, coinciding with a constant increase in oil content (from 2.8% at 150 DAFS to 11.5% at 390 DAFS) and fruit weight (Additional file 9). These results are related to the maturation process, which occur while the fruit is still in the tree, characterized by an active cell growth and cell division until the last stage of maturation [24]. Moreover, during this process of maturation and not later in ripening, the biosynthesis of fatty acids and triacylglycerols (TAGs) is being carried out [6, 18, 25].

### Gene ontology (GO) classification of DEGs during development

In order to globally comprehend the development process, differentially expressed genes were used to perform GO analyzes, using the comparisons of 150 DAFS vs 240 DAFS, 240 DAFS vs 300 DAFS and 300 DAFS vs 390DAFS. For the first comparison (150 DAFS vs 240 DAFS), we obtained an enrichment of transcripts involved in the processes of ‘lipid localization’ (GO:0010876), ‘tubulin complex’ (GO:0045298), ‘fatty-acid synthase activity’ (GO:0004312) and ‘acyl carrier activity’ (GO:0000036), involved in the fatty-acid biosynthesis (Additional file 3). In these stages, the oil content increases from 2.8 to 6% (Additional file 9), which is stored as TAG and involves the synthesis of fatty-acids (FA) in the plastid. Initially acetyl-CoA is carboxilated to form malonyl-CoA, followed by its repeated condensation with a growing acyl-carrier protein to finally obtain de novo synthesized FA. Finally, these FA are incorportated into glycerol backbones to generate TAG in the endoplasmic reticulum [26]. As previously reported in avocado, the oil content and transcript levels of acyl-carrier proteins increase representing about 24% of the total fatty-acid gene expression during maturation of the mesocarp [18].

Later in development, when comparing 240 DAFS and 300 DAFS (first harvest), we observed the overexpression of genes related to ‘response to hydrogen peroxide’ (GO:0042542) and ‘response to reactive oxygen species’ (GO:0000302), suggesting that reactive oxygen species (ROS) generated earlier in cell metabolism are being regulated during maturation (Additional file 3). Furthermore, it is known that during fruit ripening in tomato and muskmelon, there has to be a balance between the production of ROS and its removal by the action of antioxidant mechanisms [27, 28]. From these mechanisms, ascorbic acid is a powerfull antioxidant involved in the removal of ROS during cell growth, cell division, cell wall expansion and organogenesis [28–31]. In the case of avocado, this process might be occurring before fruit ripening since its development is characterized by constant cell division and cell growth.

For the third comparison during late development (300 DAFS vs 390 DAFS), genes related to ‘post-embryonic morphogenesis’ (GO:0009886) and ‘response to virus’ (GO:0009615) were overexpressed (Additional file 3). These results are associated to a full development of the seed together with an increase in susceptibility to biotic stress during ripening, since the unripe mesocarp of avocado is more tolerant to pathogen attack [6]. For example, changes related to susceptibility to pathogen infection were previously observed in tomato, were the presence of the vacuolar protease SlVPE3 is necessary for resistance against the fungal pathogen *Botrytis cinerea* during ripening [32].

### Differentially expressed genes during the fruit developmental process

Among the four developmental stages, we obtained 8672 differentially expressed genes, which were clustered in 15 groups according to their regulation pattern (Fig. 3a). In order to obtain candidate genes as potential biomarkers for the monitoring of fruit development and/or harvest date, transcripts from subcluster 4, 7 and 11 were selected due to their expression changes during the four stages of development and functional annotation was performed with BLASTx against the KEGG GENES database to identify associated pathways (Fig. 3b; Additional file 7). For these subclusters, transcripts that increase (subcluster 4) or decrease (subcluster 7 and 11) their levels were related to different processes like carotenoid biosynthesis, which is involved in the flesh color that in ‘Hass’ depends on the ripe state, changing from light green to dark green after harvest as observed in Additional file 9 [33]. The pathways of fatty acid biosynthesis and starch and sucrose metabolism were also observed, where sugars are important to promote cell growth and to control fruit metabolism during development [34]. Additionally, the phenylpropanoid biosynthesis pathway was also represented, where the secondary metabolites act as antioxidants and in tomato fruit are involved in pigmentation and aroma [35].

To obtain candidate genes for fruit development, we selected transcripts from subcluster 4 that constantly increase their expression and genes from subcluster 7 and 11 that constantly decrease their expression. For subcluster 4, we identified a homolog with similarity to oleosin (CBV_PAM10189), which usually is involved in the storage of oil bodies in seeds and pollens, however, it has also been reported its presence in avocado mesocarp, suggesting an additional role for these lipid droplet proteins during avocado fruit development [17, 36]. The homolog to beta-glucosidase 22 (CBV_PAM50790) was also present in this group and is involved in many processes of plant metabolism like the formation of intermediates for cell wall lignification and plant defense against biotic stress [37]. This is concomitant with the lignification of the endocarp for seed protection and the increasing susceptibility to pathogen infection during development [32, 38]. Additionally, the homolog of cinnamoyl-CoA reductase (CBV_PAM74617) is also associated to the lignin biosynthesis pathway. Lignin is synthesized from the phenylpropanoid pathway, where cinnamoyl-CoA reductase is a key enzyme for this process [39].

On the other side, the homolog of UDP-glucosyltransferase 85A4 (CBV_PAM23929) is involved in the transfer of glycosyl residues from nucleotide sugars to acceptor molecules and can regulate many properties of plant hormone and secondary metabolites like their bioactivity, solubility and their transport properties within the cell and throughout the plant, while zeatin O-glucosyltransferase-like (CBV_PAM38155) has a role in maintaining cytokinin homeostasis during development [40, 41]. Meantime, chitinases are pathogenesis-related proteins that are constitutively present in plants, however they can be developmentally upregulated by a growth regulator such as ethylene [42, 43]. In our case, the increase in the expression of the putative chitinase 1 (CBV_PAM2452), together with the ethylene-responsive transcription factor RAP2–4-like (CBV_PAM3819) could indicate the increase of ethylene biosynthesis, which regulates the ripening of climacteric fruit such as avocado [44]. In the case of auxins, they play a critical role during fruit development since they regulate fruit set, fruit growth and ripening, causing the increase of auxin-induced proteins like 5NG4 (CBV_PAM11370) and some ABC transporters (CBV_PAM11012) [45, 46]. Together with ethylene and auxin, transmembrane transport of gibberellin (GA) is required during plant growth and development. The coordinated work of phytohormones is crucial for fruit development, being GA an essential hormone that promotes fruit growth [47, 48].

It was previously described in *Arabidopsis thaliana* that SWEET proteins are involved in the transport of GA, indicating that the decrease of transcripts with homology to a bidirectional sugar transporter SWEET14 (CBV_PAM37547) in subcluster 7 could be associated to changes in GA transport in fruit maturity [49]. Additionally, a decrease of expression in the homolog of a Rapid Alkalinization Factor (RALF) (CBV_PAM51147) is also related to plant development and growth (Additional file 5 and Additional file 6: Table S2). RALFs are cysteine-rich peptides that allows for the communication of signaling networks across the organism, controlling processes like stem cell division, differentiation and cell expansion [50, 51]. Other transcripts that decrease their levels are ceramides, which are a family of lipid molecules that are present within the cell membrane in higher plants and their homeostasis is maintained by ceramidases [52]. In the case of avocado, as lipid metabolism is active during development, the balance of these ceramides through the sphingolipid pathway could be associated to changes in the transcript levels of neutral-ceramidases (CBV_PAM51593, CBV_PAM52108) during fruit maturation. In addition, transcripts with similarity to polyphenol oxidases (PPO) (CBV_PAM17119, CBV_PAM55044), are also downregulated during the different developmental stages since the presence of phenolic compounds are in part responsible for color, astringency, bitterness, flavor and nutritional quality in fruits [53, 54]. In relation to cell growth and division, which are constantly occurring in avocado, the coordination and regulation of these processes are important to achieve a specific shape or size. This is in part regulated by cell surface receptors known as receptor-like protein kinases, which are also decreasing their expression while the fruit is developing (CBV_PAM38418 and CBV_PAM25422) [55].

For subcluster 11, genes that constantly decrease their expression are mainly part of the cell wall structure and lipid metabolism. From these, four genes are homologs to proline-rich proteins (PRPs) (CBV_PAM10195, CBV_PAM10256, CBV_PAM1216, CBV_PAM51992), which are a principal constituent of the cell wall (Additional file 5 and Additional file 6: Table S2). The cell wall is a dynamic and complex structure that differentiates cell types during plant growth and development [56]. Our results coincide with previous reports in tomato and a higher plant like watermelon (*Citrullus lanatus*), where PRPs are highly expressed during an immature stage of fruit development that requires cell wall synthesis for cell division and cell growth, also suggesting that ethylene mediated pathways can downregulate these genes [57, 58]. From the lipid metabolism, the decreasing expression of fatty acid desaturases in avocado mesocarp was previously observed and this also agrees with the changing fatty acid composition during fruit development before harvest (pre-climacteric stage). Since no significant changes in fatty acid composition were detected during ripening, some authors hypothesize that the increase of ethylene might halt lipid biosynthesis [17, 59].

### Gene expression of genes related to fatty acid and TAG biosynthesis

Avocado fruit accumulate TAGs synthesized mainly from oleic acid, palmitic and linoleic acid [60, 61]. There are key enzymes involved in the TAG biosynthesis pathway, like acetyl-CoA carboxylase, diacylglycerol acyltransferase, several fatty acid desaturases and thioestherases [62–68]. Thus, we decided to validate the transcriptomic data using transcripts related to the TAG biosynthesis pathway (Fig. 4). Transcripts that were strongly expressed either at the start of the developmental process (150 DAFS) or at the end of the process (390 DAFS) were searched. When comparing the TPM-normalized counts with the relative abundance of transcript with qRT-PCR, the overall spearman correlation coefficient resulted over *r* = 0.85, which validates the *in-silico* assembly. We found that transcripts related to overall fatty acids and TAGs biosynthesis were upregulated at 150 DAFS and 240 DAFS, whereas downregulated in the stages of 300 DAFS and 390 DAFS. This agrees with the idea that the fruit produces the majority of its lipid content during the first half of fruit development, then those processes are not ceased but reduced [59]. In addition, oleosin was differentially expressed in mesocarp tissue between 240 DAFS to 390 DAFS, which is consistent with another study where oleosin helps to stabilize lipid storage [18].

## Conclusions

In this work, the transcript changes of the mesocarp of ‘Hass’ avocado was examined focusing in fruit development in the search for potential biomarkers for this process. Based on the annotation, it was possible to detect transcripts specifically related to lipid biosynthesis and fruit development. This transcriptomic data is consistent with previous transcriptome data generated in avocado and thus, contributes to increase the coverage data available for this economically important species. In addition, candidate genes related to lipid metabolism, ethylene signaling pathway, auxin signaling pathway, cell signaling, and components of the cell wall were identified due to their differential expression during the developing fruit, providing a foundation to further characterize these genes in the search for molecular applications. On the other hand, the main future implications of these results from an agronomical point of view, is the identification of candidate biomarkers for monitoring fruit development and harvest index, which should be validated in future work.

## Methods

### Plant material and phenotyping

‘Hass’ is the most important variety in the world representing 95% of the cultivated area, characterized by te accumulation of high levels of oil content and a quintessentially fairly thick skin that turns near black when fully ripe. From the commercial orchard located on Panquehue Valley (32°48′37.2″S; 70°50′16.7″W)″, three independent fruits (biological replicates) were sampled at 150 (2.8% oil), 240 (6% oil), 300 (7.5% oil) and 390 (11.5% oil) days after fruit set (DAFS) (Additional file 9). The oil content for each developmental stage was measured in the mesocarp by quantifying the dry matter content, considering a ~ 9% of oil content for harvest according to [69]. We determined ethylene by enclosing individual fruits in a 5000 mL jar and then sampling the air in the container. We measured ethylene by injecting 1 mL headspace sample into a gas chromatograph (GC-2014, Shimadzu, Japan) equipped with a flame ionization detector and an alumina column (Additional file 9) [70].

### RNA-Seq and de novo transcriptome assembly

RNA was extracted using RNeasy mini kit (QIAGEN, Germantown, MD, USA) from a homogenous sample of the fruit mesocarp following the manufacturer’s instruction. To verify sample integrity, total RNA was evaluated on Fragment Analyzer™ Automated CE System (Advanced Analytical Technologies, Ames, IA, USA) and quantified using Qubit® RNA BR Assay kit (Thermo Fisher Scientific). One microgram of total RNA from each sample was used as input for the Illumina® TruSeq RNA HT Sample preparation commercial kit, according with the manufacturer’s instructions. Twelve libraries (4 development stages × 3 biological replicates) were sequenced on one lane of HiSeq 2500 platform (Illumina) with reads of 2x100bp (paired-end mode).

Total reads quality parameters were analyzed (pre and post trimming) with FASTQC (https://www.bioinformatics.babraham.ac.uk/projects/fastqc/) software and the trimming process was performed using FLEXBAR software (https://github.com/seqan/flexbar) in order to filter low quality reads (phred score less than 25) and remove residual adapter sequences. Remaining reads after trimming were used as input for transcriptome assembly using Trinity software [71] with “--KMER_SIZE 20 --min_contig_length 400” [72]. After assembly, we used the CD-HIT software (http://weizhongli-lab.org/cd-hit/) to remove duplicated contigs and CORSET software (https://github.com/Oshlack/Corset/) to filter out contigs with less than 36 reads.

### Quality check and annotation of the novo transcriptome

Quality assessment was performed with two approaches: (i) a biological approach that used two ultra-conserved protein gene finder software: Core Eukaryotic Gene Mapping Approach (CEGMA) and Benchmarking Universal Single-Copy Orthologs (BUSCO) and (ii) an informatic approach, DETONATE and TransRate to assess the architecture and quality of the contigs. To perform the in-silico validation of de novo transcriptome, we used, which search ultra-conserved genes by sequence similarity on trinity genes. Annotation was performed with BLAST in BLASTx mode against the NCBI “non-redundant” protein database, reporting hits with minimum e-value of 10^− 3^ [72–75].

### Principal component analysis and differential expression analysis

A principal component analysis (PCA) and heatmap were conducted to determine grouping and similarity of each sample (biological replicates) using Trinity analysis utilities. Raw counts of each contig were obtained with RSEM using Bowtie2 aligner to align the input reads to the de novo assembled transcriptome [76, 77]. From the raw counts matrix was calculated a TPM-TMM normalized matrix used for the study alongside the raw counts matrix. Differential expression analysis was performed by using R’s package edgeR from Bioconductor. Differentially expressed genes (DEGs) were used for further comparisons and downstream analyses (False Discovery Rate; FDR < 0.01).

### Gene ontology (GO), subclustering and KEGG analysis from DEGs

DEGs were used for Singular enrichment analysis (SEA) with AgriGO v2.0 web software (http://bioinfo.cau.edu.cn/agriGO/analysis.php) and *Arabidopsis thaliana* TAIR10 as background specie [78]. DEGs-SEA data was exported to REVIGO web software (http://revigo.irb.hr/) to reduce the redundancy of GO terms (FDR < 0.05) [79].

From DEGs with at least four fold-change (log_2_(4)) and with an FDR less than 0.01, we normalized expression values and grouped them in similar expression patterns. Thus, we generated a heatmap using the ggplot2 package, which shows the relationships between genes and samples. Then, to generate the subclusters we extracted the hierarchical grouping obtained earlier considering K equal to 15 using the MeV software. From the clustering data, FASTA nucleotide sequences were obtained from the transcripts present in subclusters 4, 7 and 11 and loaded into KAAS (KEGG Automatic Annotation Server; https://www.genome.jp/kegg/kaas/) to provide functional annotation of genes that constantly increase/decrease their transcript levels.

### RNA-seq validation through qRT-PCR analysis

In order to perform a technical validation of DEG analysis, we designed primers using the software Primer 3 Plus (http://www.bioin formatics.nl/cgi-bin/primer3plus/primer3plus.cgi) based on the sequences generated by the de novo assembly. First, from the same input used to construct the libraries, total RNA was treated with DNase I (Fermentas, Thermo Fisher Scientific, Waltham, MA, USA) according to the standard protocol. The first strand cDNA was obtained by reverse transcription using the system MMLV-RT reverse transcriptase (Promega, Madison, WI, USA) and oligo dT primers. The cDNA concentration was obtained by measuring absorbance at 260 nm. Each cDNA sample was diluted to 50 ng*μL^− 1^ before being used in qRT-PCR assays. The qRT-PCR assay was performed using a LightCycler® 96 Real-Time PCR kit (Roche Diagnostics, Mannheim, Germany) with LC-FastStart DNA Master SYBR Green I to measure the DNA product derived from RNA, as previously described by García-Rojas and colleages [80]. Additionally, to validate the gene expression profiles in the selected subclusters, qRT-PCR of one gene belonging to each subcluster (4, 7 and 11) was performed using total RNA from five independent fruits at 150, 240, 300 and 390 DAFS. All qRT-PCR were performed using five biological replicates and the gene expression values were normalized relative to the *PamActin* gene (GenBank JN786942).

## Additional files

Additional file 1: Sequencing metrics for 12 cDNA libraries, which were sequenced on one lane of HiSeq2000 (2x100bp). We showed number of raw and processed reads, quality reads and mapped reads. (DOCX 14 kb)

Additional file 2: Functional annotation of the whole de novo transcriptome of *Persea Americana* cv. Hass during fruit developmental stages. Trinity contigs were annotated against the nr database using Blastx (10^− 3^). (XLSX 4991 kb)

Additional file 3: Gene Ontology (GO) term comparisons among fruit developmental stages (150, 240, 300 and 390 days after fruit set; DAFS). GO analysis was performed using Differentally Expressed Genes (DEG; FDR < 0.01) from comparisons among 150 DAFS vs 240 DAFS, 240 DAFS vs 300 DAFS and 300 DAFS vs 390 DAFS by AgriGO v2.0 web software. (XLSX 36 kb)

Additional file 4: List of transcription factors differentally expressed during fruit development in *Persea Americana* cv. Hass. Transcription factors were selected using Differentally Expressed Genes (DEG; FDR < 0.01) from comparisons among 150 DAFS vs 240 DAFS, 240 DAFS vs 300 DAFS and 300 DAFS vs 390 DAFS. (XLSX 23 kb)

Additional file 5: Expression subcluster matrix. Values of Trinity gene expression (TPM-TMM) for 15 subclusters identified by gene expression cluster analysis during fruit developmental stages (150, 240, 300 and 390 days after fruit set; DAFS) in *Persea americana* cv. Hass. (XLSX 713 kb)

Additional file 6: Expression subcluster plots. Analysis of gene expression cluster during fruit developmental stages (150, 240, 300 and 390 days after fruit set; DAFS) in *Persea americana* cv. Hass. (PDF 762 kb)

Additional file 7: KEGG pathway analysis from selected subclusters 4, 7 and 11 from gene expression cluster analysis. Using Differentally Expressed Genes (DEG; FDR < 0.01) from comparisons among 150 DAFS (Days After Fruit Set) vs 240 DAFS, 240 DAFS vs 300 DAFS and 300 DAFS vs 390 DAFS. (DOCX 290 kb)

Additional file 8: List of the primers used for validated gene expression levels by qRT-PCR. Trinity genes were validated by qRT-PCR assays and the correlation between gene expression level obtained by qRT-PCR and RNA-Seq data are shown. (DOCX 15 kb)

Additional file 9: Fruit developmental stages phenotyping of *Persea americana* cv. ‘Hass’. we phenotyped fruit weight (g), ethylene production (uL C_2_H_4_kg^− 1^ h^− 1^) and oil content (5) for fruit developmental stages during 150, 240, 300 and 390 days after fruit set. (DOCX 1082 kb)

## Abbreviations

BLAST: Basic Local Alignment Search Tool
BUSCO: Benchmarking Universal Single-Copy Orthologs
CEGMA: Core Eukaryotic Genes Mapping Approach
DAFS: Days After Fruit Set
DEGs: Differentially Expressed Genes
DETONATE: DE novo TranscriptOme rNa-seq Assembly
FC: Fold Change
FDR: False Discovery Rate
GA: Gibberellin
GO: Gene Ontology
MAS: Marker-Assisted Selection
MMLV-RT: Moloney Murine Leukemia Virus Reverse Transcriptase
NGS: Next-Generation Sequencing
PCA: Principal Component Analysis
PPO: Polyphenol Oxidases
PRPs: Proline-Rich Proteins
RALF: Rapid Alkalinization Factor
REVIGO: Reduce + Visualize Gene Ontology
ROS: Reactive Oxygen Species
SEA: Singular Enrichment Analysis
TAGs: Triacylglycerols
TAIR: The Arabidopsis Information Resource
TMM: Trimmed Mean of M-values
TPM: Transcripts Per Million
