# Supplementary material for: De novo assembly of Persea americana cv. ‘Hass’ transcriptome during fruit development
Source: BMC Genomics. 2019 Feb 6;20:108. doi: 10.1186/s12864-019-5486-7 (PMC6364401; doi:10.1186/s12864-019-5486-7)
Supplement: Supplementary file 1 — Sequencing metrics for 12 cDNA libraries, which were sequenced on one lane of HiSeq2000 (2x100bp). We showed number of raw and processed reads, quality reads and mapped reads. (DOCX 14 kb) [file 12864_2019_5486_MOESM1_ESM.docx]

**Additional file 2.** Sequencing metrics for 12 cDNA libraries, which were sequenced on one lane of HiSeq2000 (2x100bp. Number of raw and processed reads, quality reads and mapped reads are shown.

| Mesocarp library | Raw reads | Processed reads | Q20 (%) | Q30 (%) | Mapped reads (%) |
| --- | --- | --- | --- | --- | --- |
| 150DAFS R1 | 24,820,840 | 24,801,818 | 96.3 | 94.1 | 97.1 |
| 150DAFS R2 | 27,616,648 | 27,595,446 | 96.4 | 94.2 | 96.4 |
| 150DAFS R3 | 19,649,936 | 19,634,482 | 96.4 | 94.2 | 96.8 |
| 240DAFS R1 | 19,616,648 | 19,615,054 | 96.4 | 94.2 | 96.2 |
| 240DAFS R2 | 19,639,130 | 19,032,770 | 96.5 | 94.3 | 96.4 |
| 240DAFS R3 | 24,149,248 | 24,130,282 | 96.5 | 94.3 | 96.2 |
| 300DAFS R1 | 20,563,966 | 20,547,914 | 96.4 | 94.2 | 96.3 |
| 300DAFS R2 | 22,663,706 | 22,645,560 | 96.5 | 94.3 | 96.1 |
| 300DAFS R3 | 21,796,710 | 21,780,054 | 96.4 | 94.1 | 96.3 |
| 390DAFS R1 | 21,191,950 | 21,175,460 | 96.3 | 95.0 | 96.9 |
| 390DAFS R2 | 19,394,744 | 19,379,890 | 96.4 | 94.2 | 96.8 |
| 390DAFS R3 | 20,305,700 | 20,290,018 | 96.5 | 94.4 | 95.1 |
| Total | 261,409,226 | 260,628,748 | 96.5 | 94.3 | 94.1 |

DAFS: Days after fruit set; Q20: Phred quality score over 20; Q30: Phred quality score over 30.
