# Supplementary material for: De novo assembly of Persea americana cv. ‘Hass’ transcriptome during fruit development
Source: BMC Genomics. 2019 Feb 6;20:108. doi: 10.1186/s12864-019-5486-7 (PMC6364401; doi:10.1186/s12864-019-5486-7)
Supplement: Supplementary file 6 — Expression subcluster plots. Analysis of gene expression cluster during fruit developmental stages (150, 240, 300 and 390 days after fruit set; DAFS) in Persea americana cv. Hass. (PDF 762 kb) [file 12864_2019_5486_MOESM6_ESM.pdf]

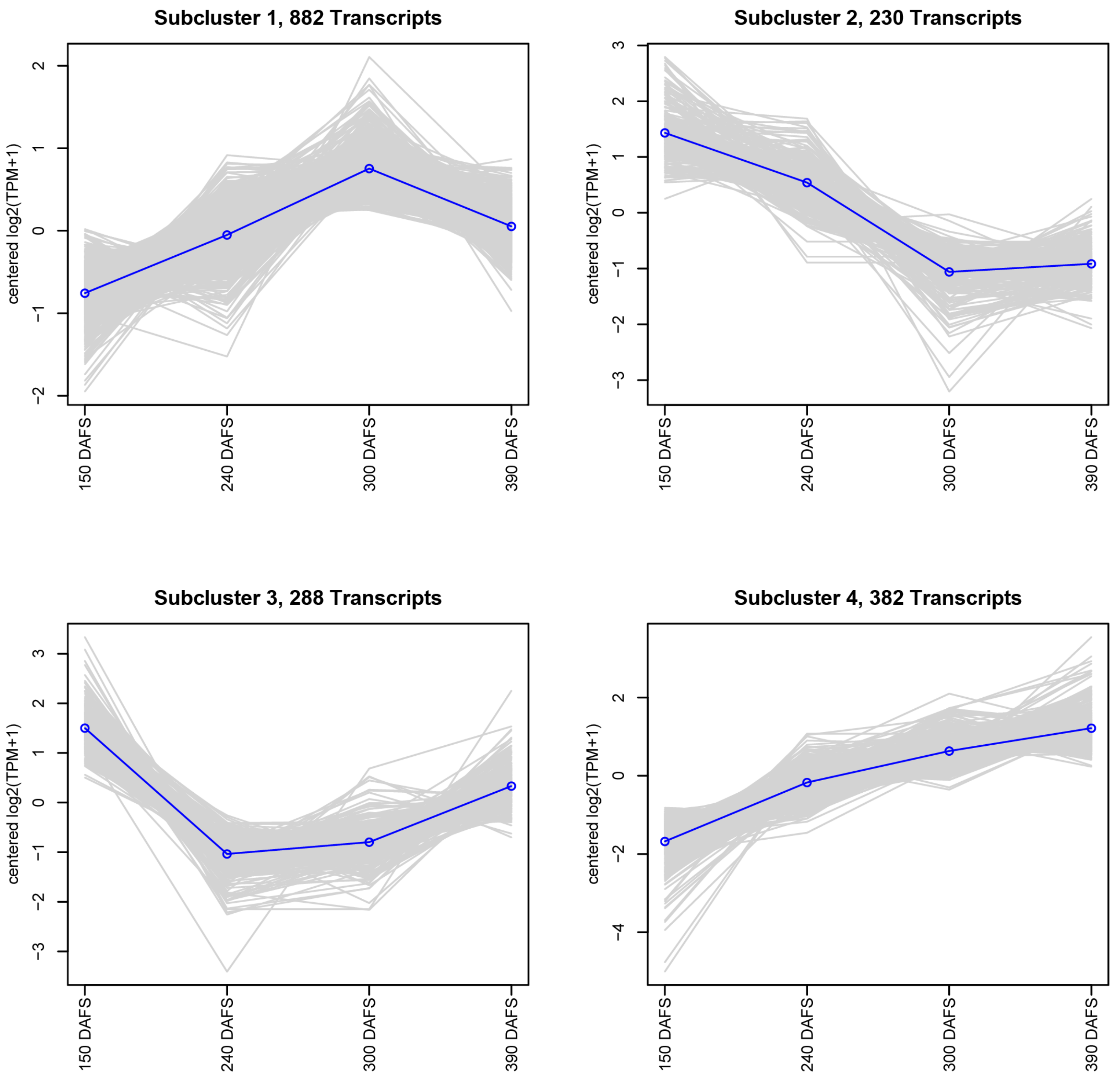
Subcluster 1, 882 Transcripts
Subcluster 2, 230 Transcripts
Subcluster 3, 288 Transcripts
Subcluster 4, 382 Transcripts
centered log2(TPM+1)
150 DAFS
240 DAFS
300 DAFS
390 DAFS

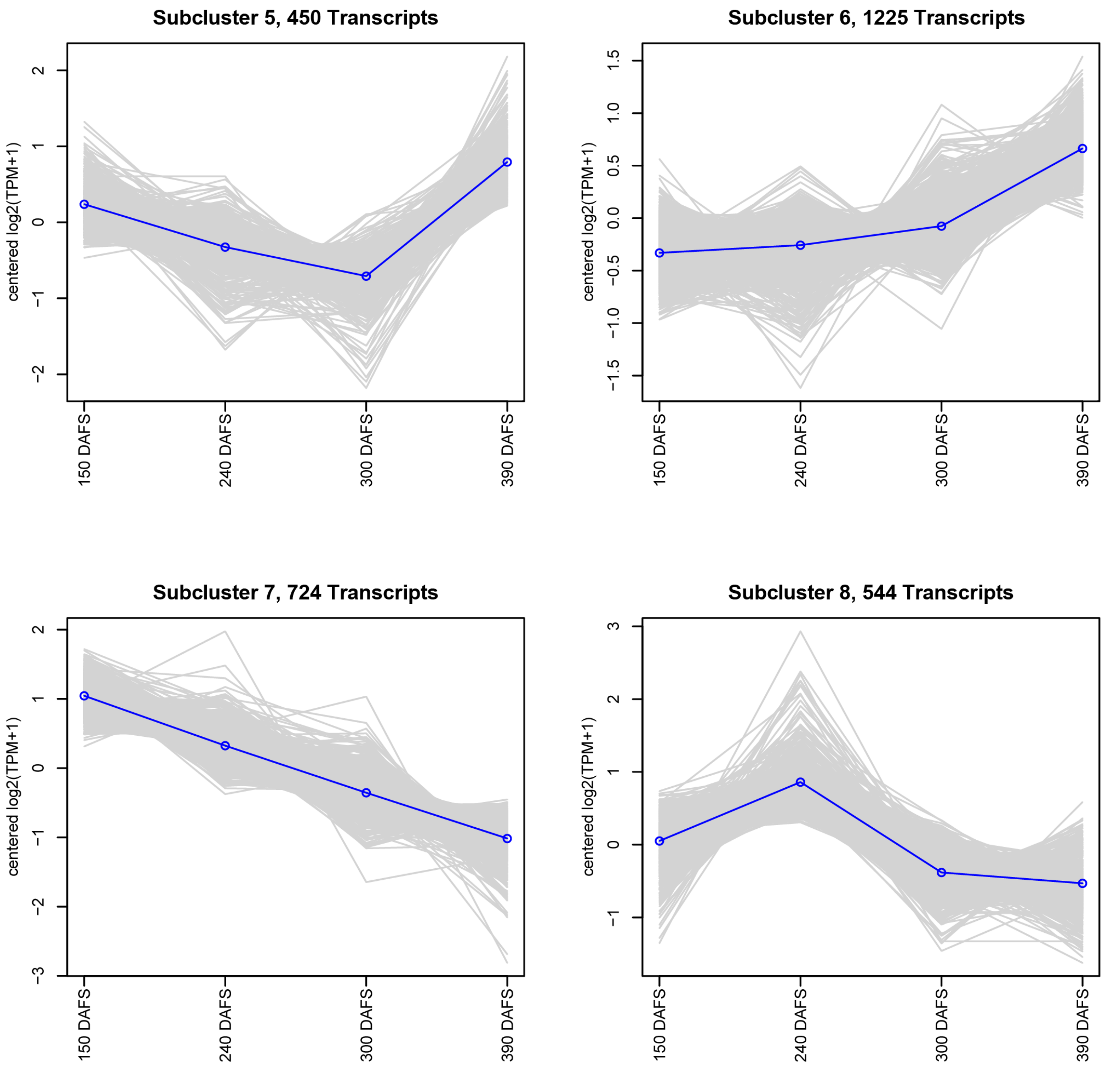
Subcluster 5, 450 Transcripts
Subcluster 6, 1225 Transcripts
Subcluster 7, 724 Transcripts
Subcluster 8, 544 Transcripts
centered log2(TPM+1)
150 DAFS
240 DAFS
300 DAFS
390 DAFS

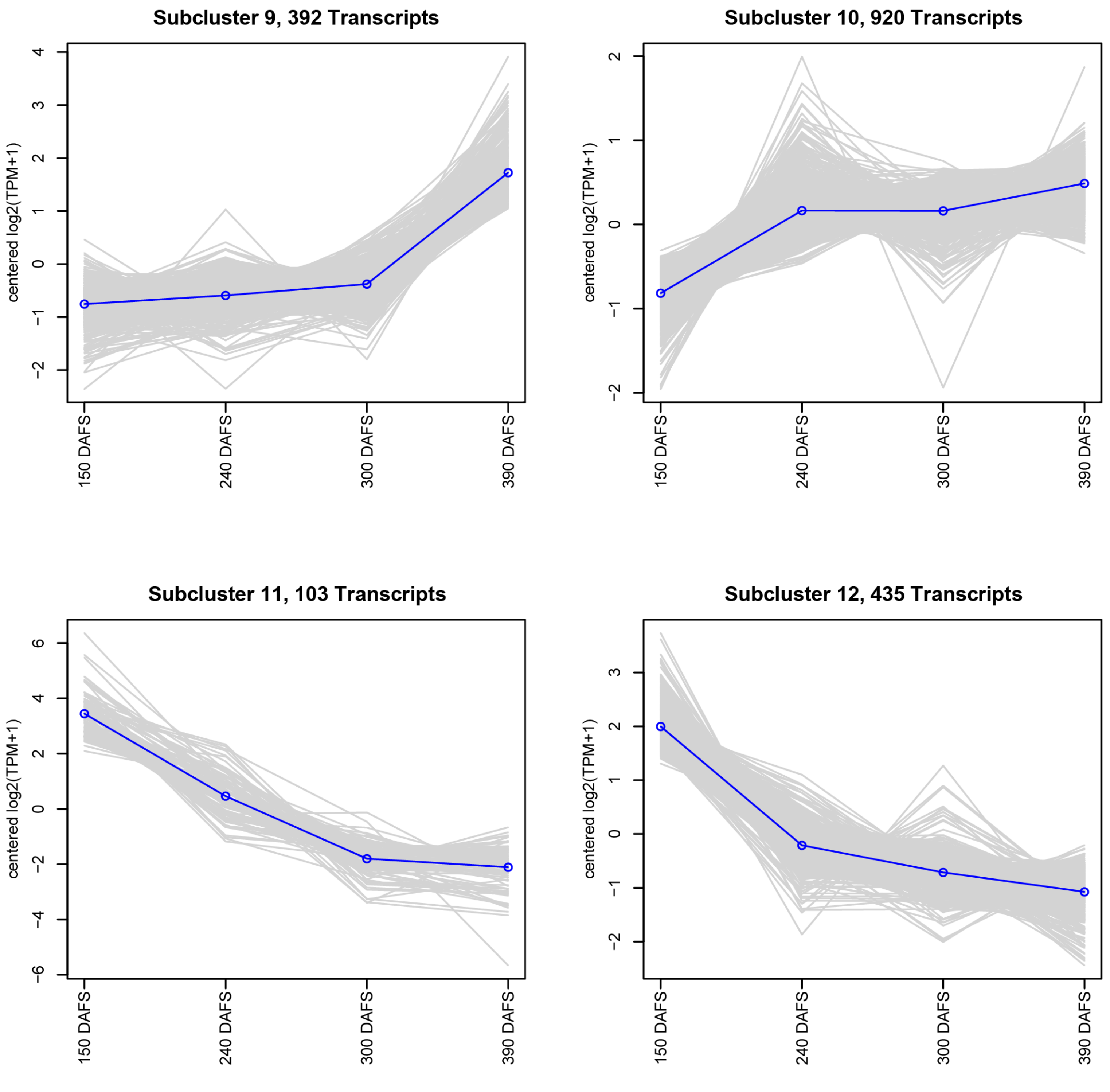
Subcluster 9, 392 Transcripts
Subcluster 10, 920 Transcripts
Subcluster 11, 103 Transcripts
Subcluster 12, 435 Transcripts
centered log2(TPM+1)
150 DAFS
240 DAFS
300 DAFS
390 DAFS

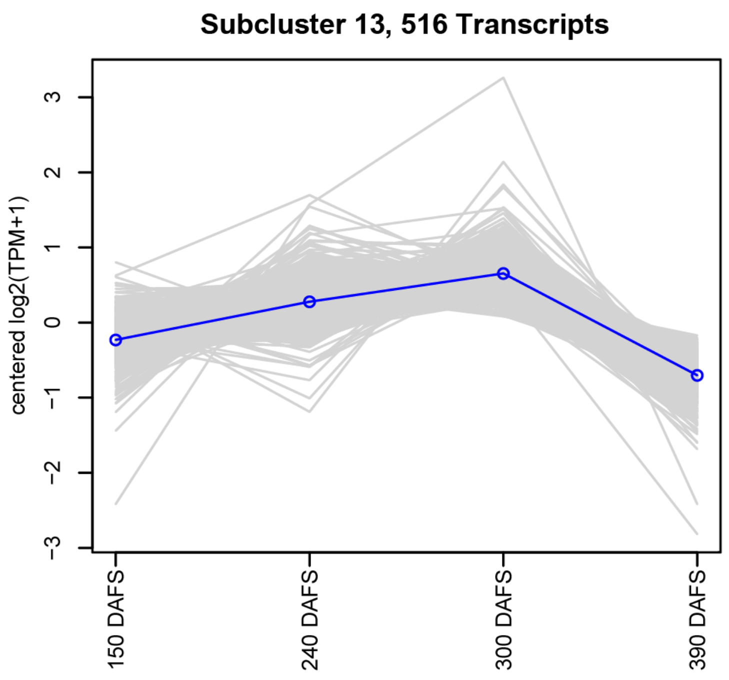
Subcluster 13, 516 Transcripts
centered log2(TPM+1)
3
2
1
0
−1
−2
−3
150 DAFS
240 DAFS
300 DAFS
390 DAFS

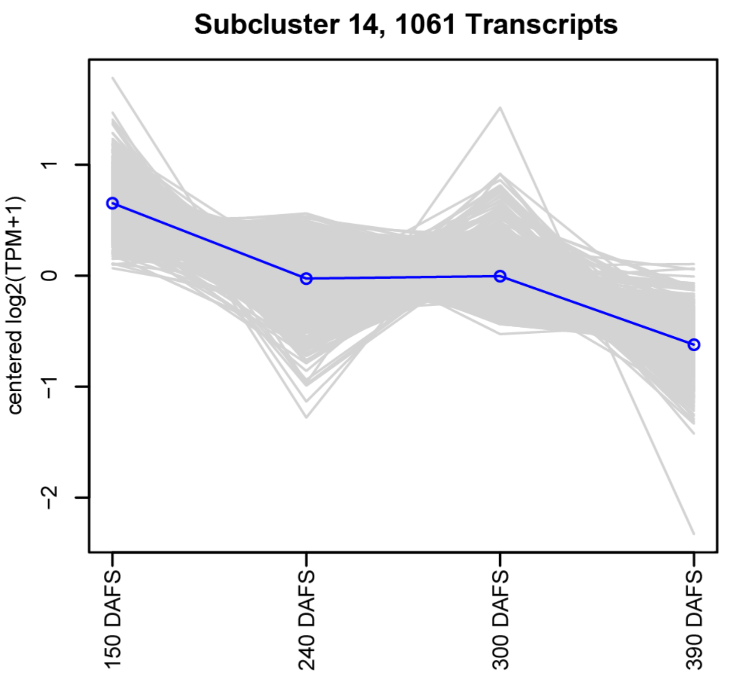
Subcluster 14, 1061 Transcripts
centered log2(TPM+1)
1
0
−1
−2
150 DAFS
240 DAFS
300 DAFS
390 DAFS

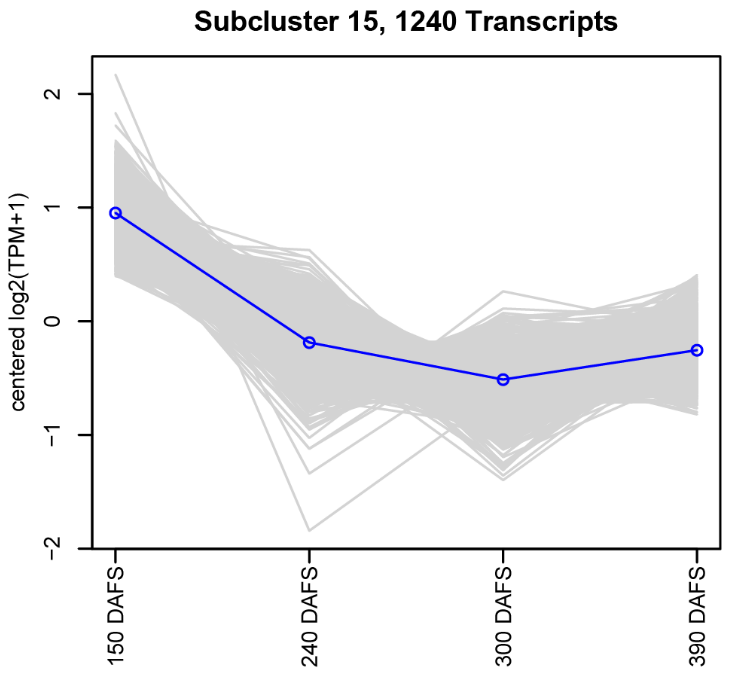
Subcluster 15, 1240 Transcripts
centered log2(TPM+1)
2
1
0
−1
−2
150 DAFS
240 DAFS
300 DAFS
390 DAFS
