# Supplementary material for: De novo assembly of Persea americana cv. ‘Hass’ transcriptome during fruit development
Source: BMC Genomics. 2019 Feb 6;20:108. doi: 10.1186/s12864-019-5486-7 (PMC6364401; doi:10.1186/s12864-019-5486-7)
Supplement: Supplementary file 7 — KEGG pathway analysis from selected subclusters 4, 7 and 11 from gene expression cluster analysis. Using Differentally Expressed Genes (DEG; FDR < 0.01) from comparisons among 150 DAFS (Days After Fruit Set) vs 240 DAFS, 240 DAFS vs 300 DAFS and 300 DAFS vs 390 DAFS. (DOCX 290 kb) [file 12864_2019_5486_MOESM7_ESM.docx]

**Additional file 8.** KEGG pathway analysis from selected subclusters 4, 7 and 11 from gene expression cluster analysis. Using Differentially Expressed Genes (DEG; FDR<0.01) from comparisons among 150 DAFS (Days After Fruit Set) vs 240 DAFS, 240 DAFS vs 300 DAFS and 300 DAFS vs 390 DAFS.


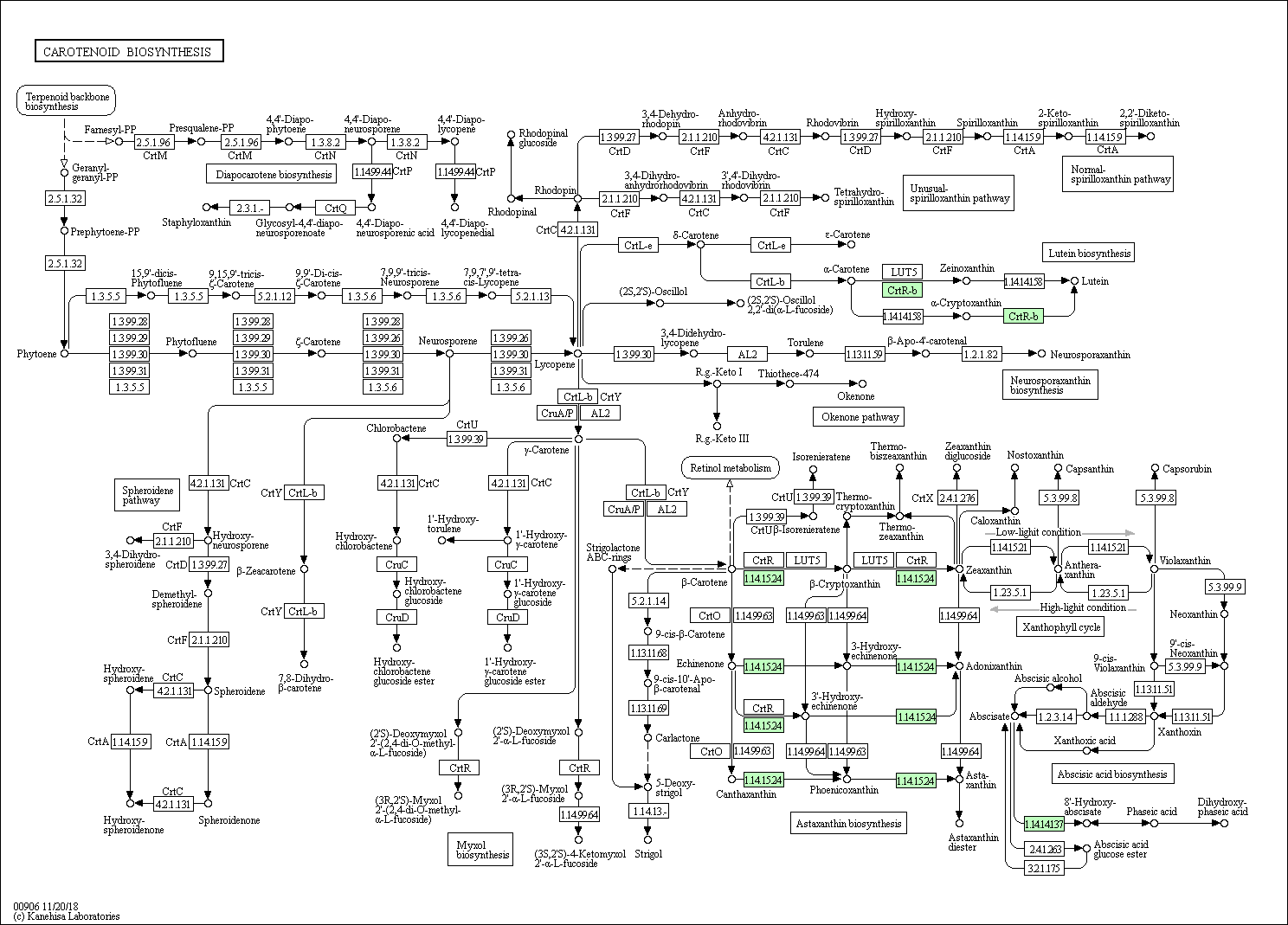


**Additional file 8.1 Carotenoid biosynthesis pathway with transcripts from subcluster 4.** Enzymes and products are represented as rectangles and circles, respectively. The presence of transcripts from subcluster 4 are indicated in green, where CrtR-b: beta-carotene 3-hydroxylase; 1.14.15.24: beta-carotene 3-hydroxylase and 1.14.14.137: (+) – abscisic acid 8’-hydroxylase.


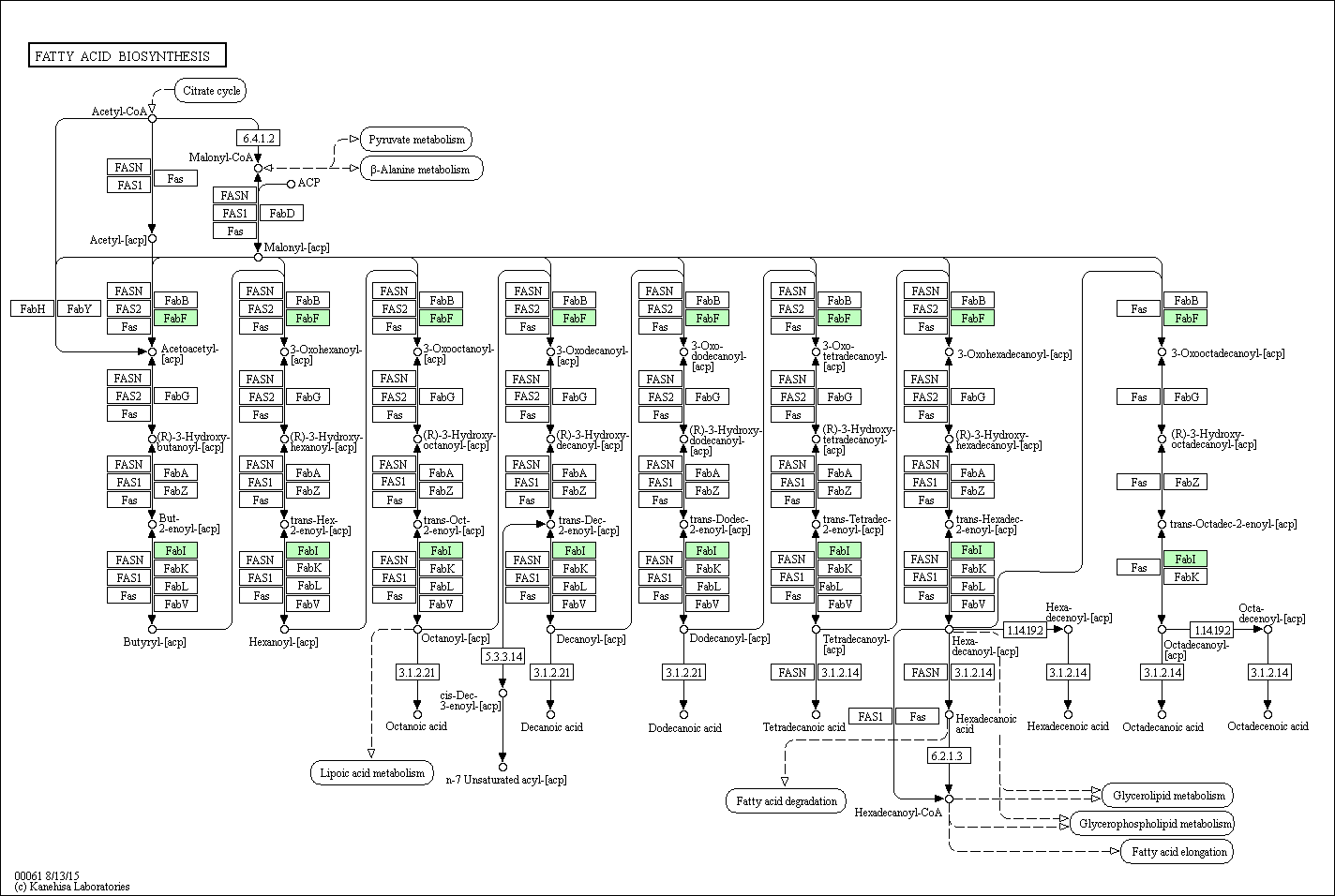


**Additional file 8.2. Fatty acid biosynthesis pathway with transcripts from subcluster 4.** Enzymes and products are represented as rectangles and circles, respectively. The presence of transcripts from subcluster 4 are indicated in green, where FabF: 3-oxoacyl-[acyl-carrier protein] synthase II and FabI: enoyl-[acyl-carrier protein] reductase I.


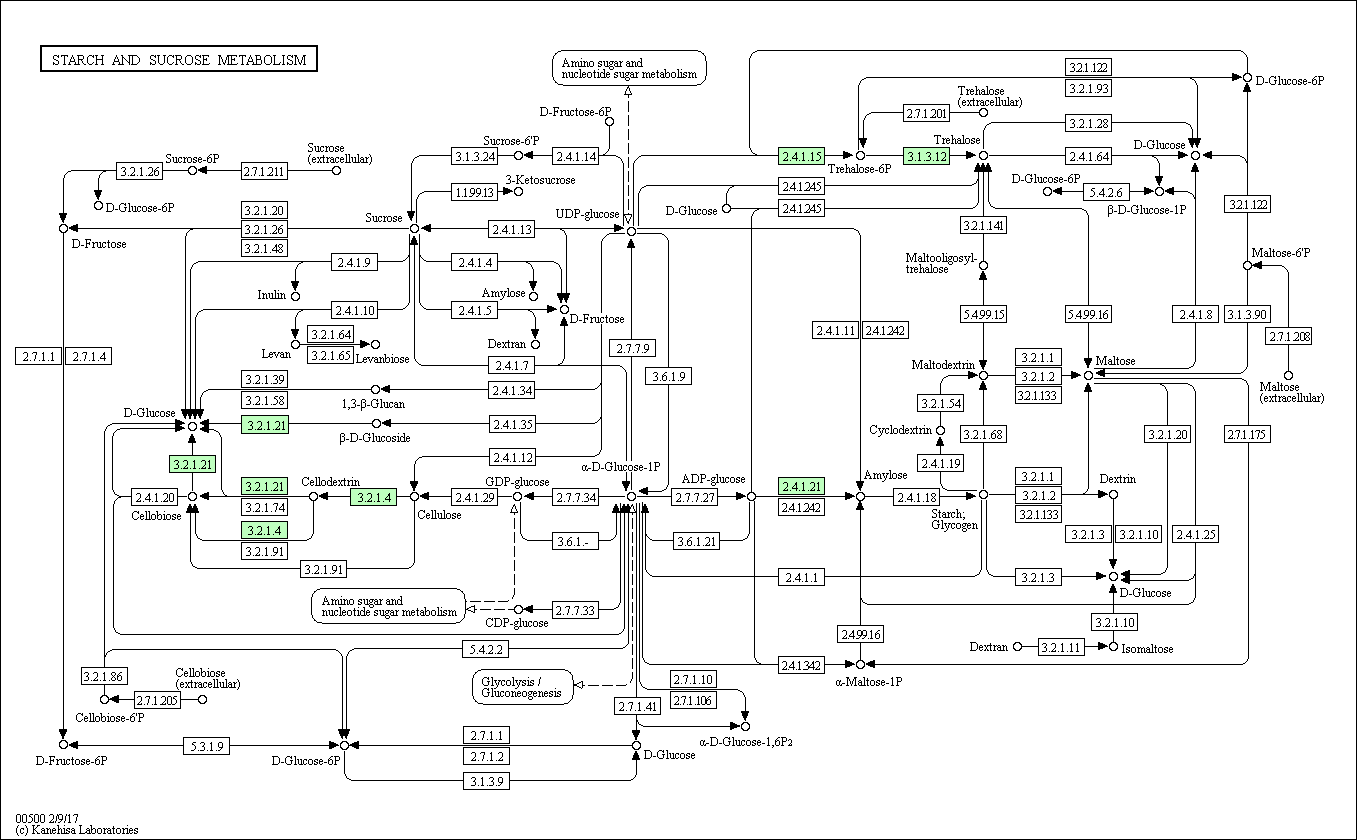


**Additional file 8.3. Starch and sucrose metabolism pathway with transcripts from subcluster 4.** Enzymes and products are represented as rectangles and circles, respectively. The presence of transcripts from subcluster 4 are indicated in green, where 2.4.1.15: trehalose 6-phosphate synthase; 3.1.3.12: trehalose 6-phosphate phosphatase; 3.2.1.21: beta-glucosidase; 3.2.1.4: endoglucanase and 2.4.1.21: starch synthase.


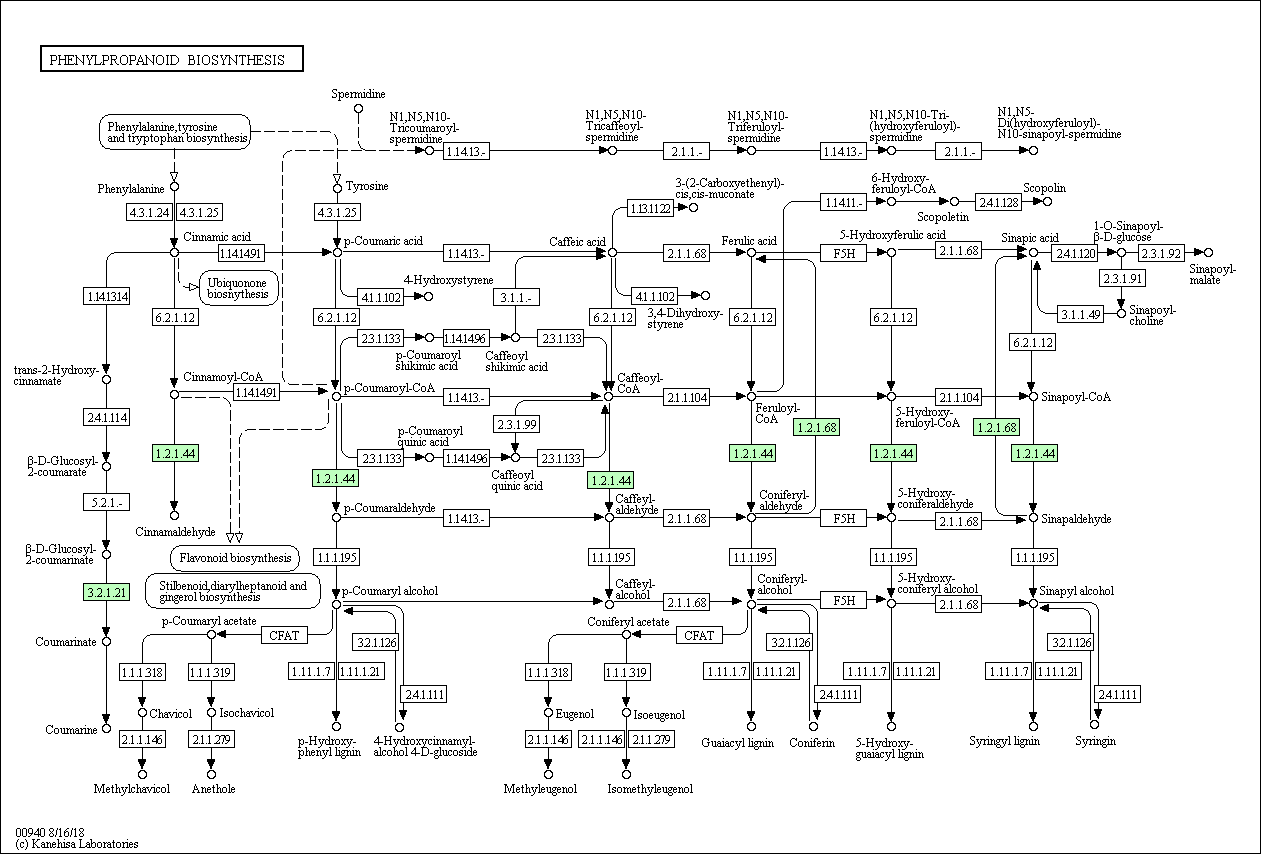


**Additional file 8.4. Phenylpropanoid biosynthesis pathway with transcripts from subcluster 4.** Enzymes and products are represented as rectangles and circles, respectively. The presence of transcripts from subcluster 4 are indicated in green, where 3.2.1.21: beta-glucosidase; 1.2.1.44: cinnamoyl-CoA reductase and 1.2.1.68: coniferyl-aldehyde dehydrogenase.


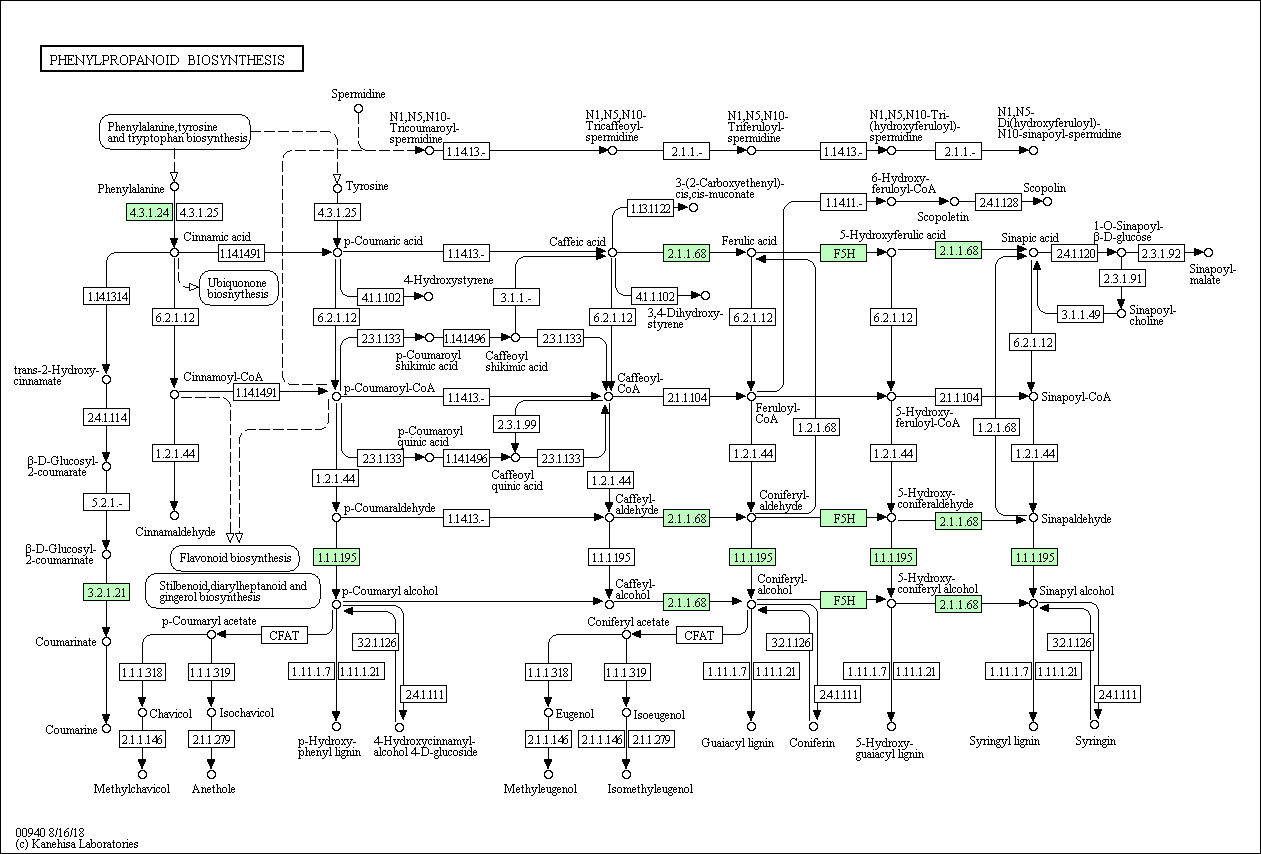


**Additional file 8.5. Phenylpropanoid biosynthesis pathway with transcripts from subcluster 7.** Enzymes and products are represented as rectangles and circles, respectively. The presence of transcripts from subcluster 7 are indicated in green, where 4.3.1.24: phenylalanine ammonia-lyase; 3.2.1.21: beta-glucosidase; 1.1.1.195: cinnamyl-alcohol dehydrogenase; 2.1.1.68: caffeic acid 3-0-methyltransferase and F5H: ferulate-5-hydroxylase.


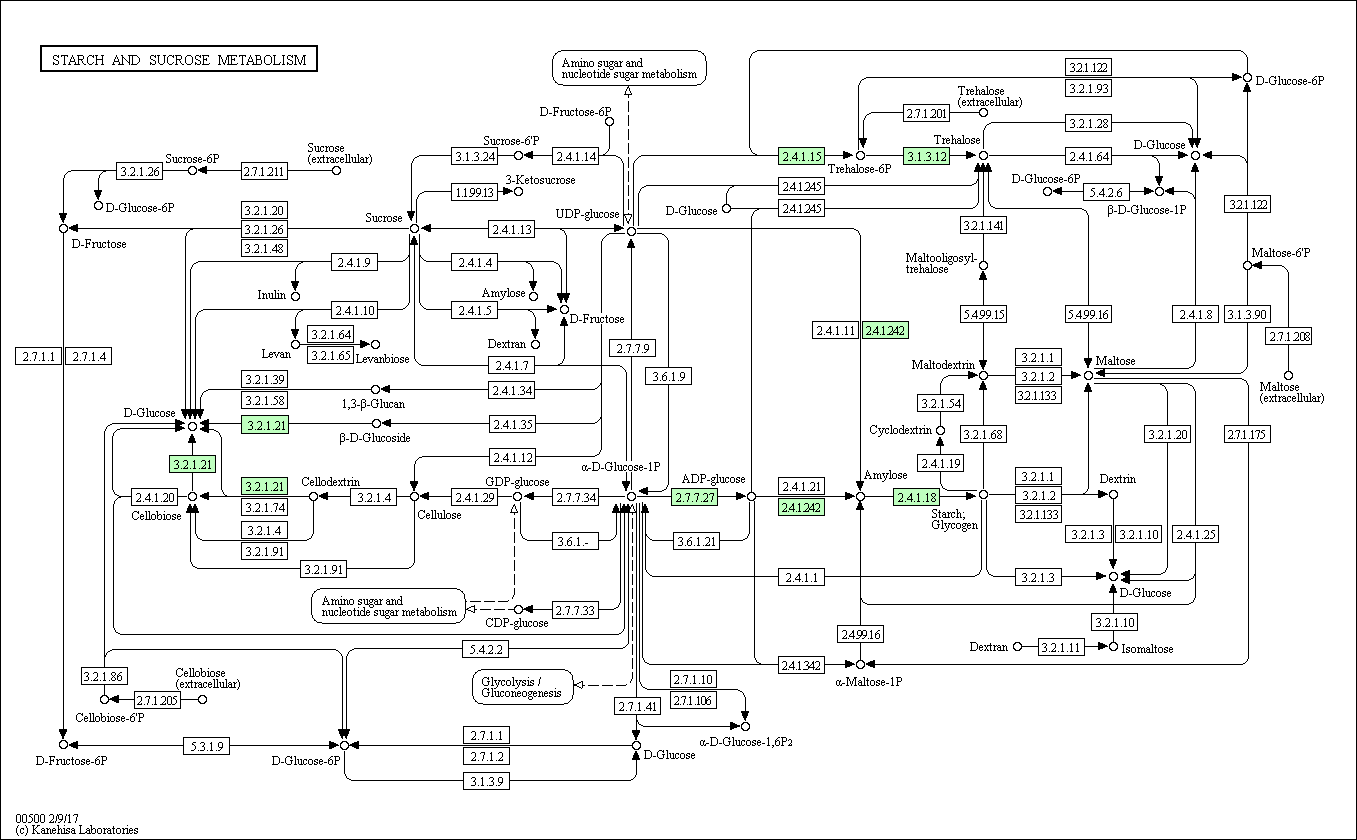


**Additional file 8.6. Starch and sucrose metabolism pathway with transcripts from subcluster 7.** Enzymes and products are represented as rectangles and circles, respectively. The presence of transcripts from subcluster 7 are indicated in green, where 3.2.1.21: beta-glucosidase; 2.7.7.27: glucose-1-phosphate adenylyltransferase; 2.4.1.242: granule-bound starch synthase and 2.4.1.18: 1,4-alpha-glucan branching enzyme.


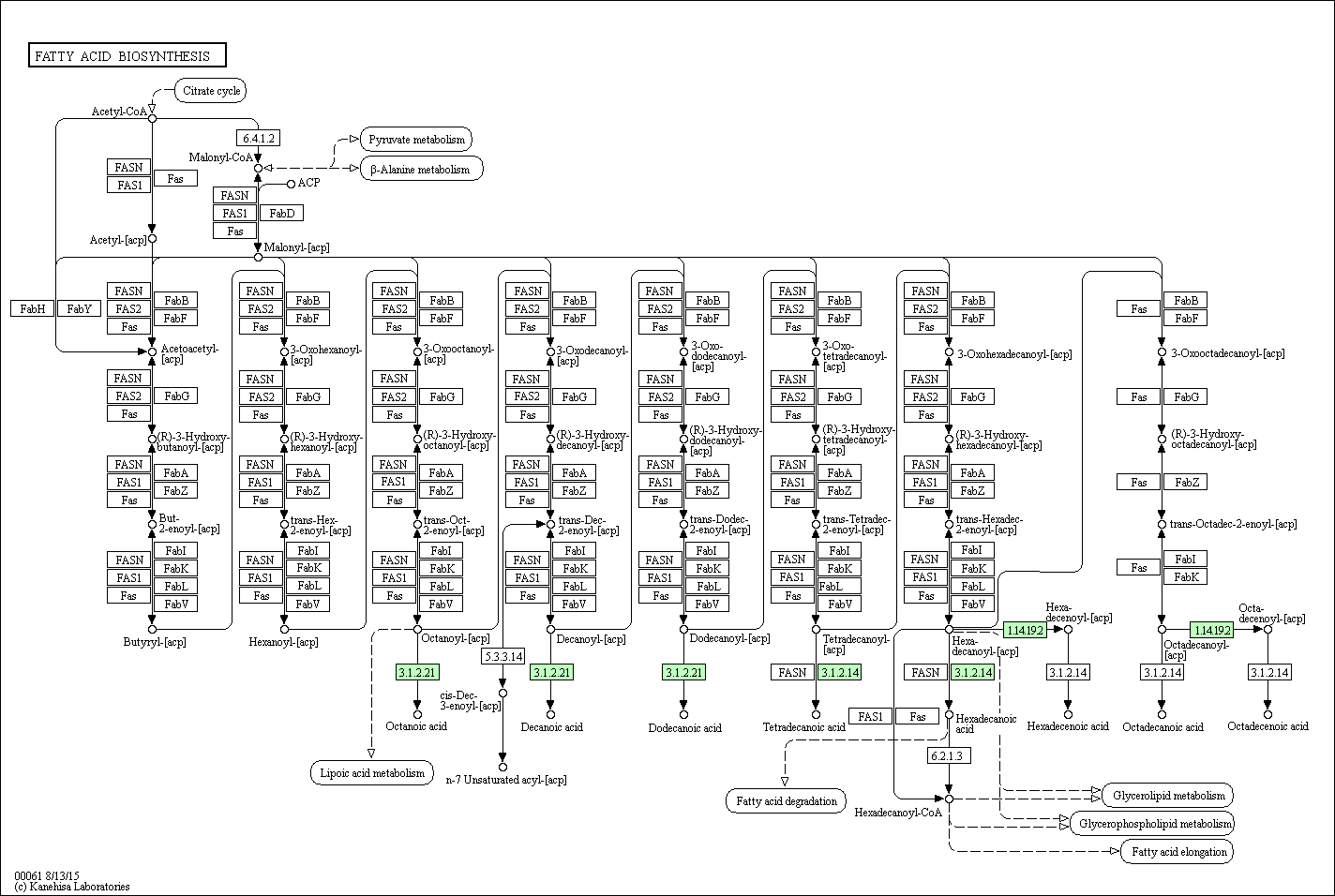


**Additional file 8.7. Fatty acid biosynthesis pathway with transcripts from subcluster 11.** Enzymes and products are represented as rectangles and circles, respectively. The presence of transcripts from subcluster 11 are indicated in green, where 3.1.2.21: medium.chain acyl-[acyl-carrier-protein] hydrolase; 3.1.2.14: fatty acyl-ACP thioesterase B and 1.14.19.2: acyl-[acyl-carrier-protein] desaturase.
