# Supplementary material for: De novo assembly of Persea americana cv. ‘Hass’ transcriptome during fruit development
Source: BMC Genomics. 2019 Feb 6;20:108. doi: 10.1186/s12864-019-5486-7 (PMC6364401; doi:10.1186/s12864-019-5486-7)
Supplement: Supplementary file 8 — List of the primers used for validated gene expression levels by qRT-PCR. Trinity genes were validated by qRT-PCR assays and the correlation between gene expression level obtained by qRT-PCR and RNA-Seq data are shown. (DOCX 15 kb) [file 12864_2019_5486_MOESM8_ESM.docx]

**Additional file 9.** List of the primers used for validated gene expression levels by qRT-PCR. Trinity genes were validated by qRT-PCR assays and the correlation between gene expression level obtained by qRT-PCR and RNA-Seq data are shown.

| Annotation | r* | Primers |
| --- | --- | --- |
| Stearoyl-(ACP) desaturase | 0.90 | F 5´ TCTCCGAGGGTTTTCATTGGC 3´  R 5´ CCGCCCAATCTTCCAAGGAT 3´ |
| β-Ketoacyl-(ACP) synthase III | 0.97 | F 5´ TCGGCACCAATTACTAGAACA 3´  R 5´ CTGCTGTGCACATCTACTCC 3´ |
| G3P-2-O-acyltransferase 6 | 0.91 | F 5´ CGAACGCGTTGAAGTGCA 3´  R 5´ GTCACTGCCAACCCCAGAAT 3´ |
| 3-Oxoacyl-(ACP) synthase I | 0.86 | F 5´ CCCCTTCCCCCATGACAAAA 3´  R 5´ TGGGACCAAACTACTCCATTTCA 3´ |
| Oleosin | 0.87 | F 5´ ATCTGTTGGGCCATGTCTCG 3´  R 5´ TCTTCTCCAGTGGGTTTGGC 3´ |
| Stearoyl-(ACP) 9-desaturase 6 | 0.98 | F 5´ CTGAAGCCGGTCGACCAG 3´  R 5´ GTGATCATGTCCCCCACCAG 3´ |
| β-D-Galactosidase | 0.96 | F 5´ TGGACTTCCTGGTTTACGGC 3´  R 5´ GTCGCAACAAAAGGACCACC 3´ |
| Glycosyl hydrolase isoform 2 | 0.94 | F 5´ GCACCATGCATTGCGGT 3´  R 5´ ACCTTTTTCTTTCCACCAACA 3´ |
| Phosphatidate phosphatase | 0.74 | F 5´ CTCTATTCCCAAAGCCCGCA 3´  R 5´ TCTGAAACAGGATGGAAAAGC 3´ |
| Oleoyl-(ACP) thioesterase 1 | 0.82 | F ´ TGCAGGAAGTTGGATGCA 3´  R ´ TGCAGGAAGTTGGATGCA 3´ |
| Enoyl-(ACP) reductase 1 | 0.77 | F 5´ GCAACACGCGTGATTCATCA 3´  R 5´ AGGCAATGTCTGGAACAAGTGA 3´ |
| Diacylglycerol acyltransferase 1 | 0.87 | F 5´ GAGATGAGGCAAGGATCGGG 3´  R 5´ AGCCTGCTATTCACCGCAAT 3´ |
| Lipid phosphate phosphatase 2 | 0.95 | F 5´ CCAGACTTCTACTGGCGTTGT 3´  R 5´ CTGATTTTCCTGACAAGTACCA 3´ |

()^*^Spearman correlation between qRT-PCR and RNA-Seq data. R: reverse primer, F: forward primer.
