# Supplementary material for: De novo assembly of Persea americana cv. ‘Hass’ transcriptome during fruit development
Source: BMC Genomics. 2019 Feb 6;20:108. doi: 10.1186/s12864-019-5486-7 (PMC6364401; doi:10.1186/s12864-019-5486-7)
Supplement: Supplementary file 9 — Fruit developmental stages phenotyping of Persea americana cv. ‘Hass’. we phenotyped fruit weight (g), ethylene production (uL C2H4kg− 1 h− 1) and oil content (5) for fruit developmental stages during 150, 240, 300 and 390 days after fruit set. (DOCX 1082 kb) [file 12864_2019_5486_MOESM9_ESM.docx]

**Additional file 1.** Fruit developmental stages phenotyping of *Persea americana* cv. ‘Hass’. Fruit weight (g), ethylene production (uL C_2_H_4_kg^-1^h^-1^) and oil content (5) for fruit developmental stages during 150, 240, 300 and 390 days after fruit set were phenotyped.

|  |  |  |
| --- | --- | --- |
|  | 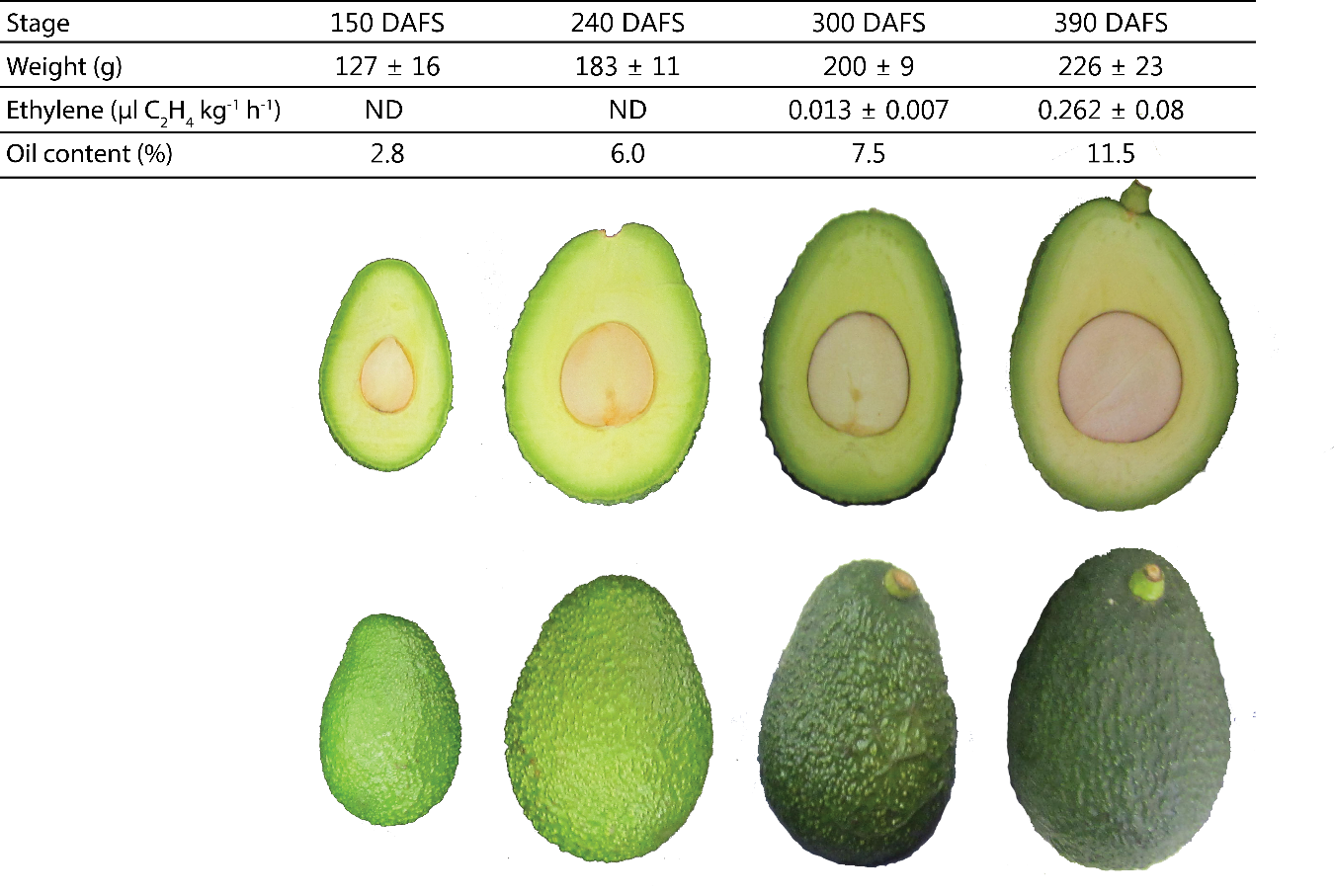 |  |
|  |  |  |
